# PRAG1 Condensation Drives Cell Contraction Under Stress

**DOI:** 10.3390/biom15030379

**Published:** 2025-03-05

**Authors:** Peiwu Ye, Peiran Jiang, Luyu Ye, Min Liu, Qiuyuan Fang, Peilin Yu, Jianhong Luo, Huanxing Su, Wei Yang

**Affiliations:** 1 Institute of Chinese Medical Sciences, University of Macau, Macau 999078, China; yc07526@umac.mo; 2 Department of Neurosurgery of the Fourth Affiliated Hospital, Zhejiang University School of Medicine, Hangzhou 310058, China; 21718539@zju.edu.cn; 3 Department of Neurobiology of the Second Affiliated Hospital, Zhejiang University School of Medicine, Hangzhou 310058, China; 4 Department of Biophysics, Institute of Neuroscience, NHC and CAMS Key Laboratory of Medical Neurobiology, Zhejiang University School of Medicine, Hangzhou 310058, China; yeluyu@zju.edu.cn (L.Y.); 12118070@zju.edu.cn (M.L.); 12018376@zju.edu.cn (Q.F.); 5 Department of Toxicology, School of Public Health, Zhejiang University School of Medicine, Hangzhou 310058, China; yupeilin@zju.edu.cn; 6 School of Brain Science and Brain Medicine, Zhejiang University, Hangzhou 310058, China; luojianhong@zju.edu.cn; 7 Guizhou University Medical College, Guiyang 550025, China

**Keywords:** PRAG1, condensation, phase separation, cell contraction, stress

## Abstract

Peak1-related, kinase-activating pseudokinase 1 (PRAG1), a member of the pseudopodium-enriched atypical kinase (PEAK) family of pseudokinases, has been reported to play a role in regulating cell morphology. However, the molecular mechanism for this function remains elusive. In this study, we demonstrate that PRAG1 forms dynamic condensates in cells mediated by its αN and αJ helices. Importantly, we found that PRAG1 condensates functioned in mediating cell contraction, while condensate-formation-deficient PRAG1 mutants lost this function. Remarkably, the formation of spherical PRAG1 condensates appears to be a common phenomenon in diverse stress models, as well as in dopaminergic (DA) neurons derived from a Parkinson’s disease patient. Our findings reveal a novel mechanism through which PRAG1 drives cell contraction and suggest a potential link between aberrant PRAG1 phase separation and stress-induced cell contraction. PRAG1 condensation drives cell contraction under stress.

## 1. Introduction

Although pseudokinases lack kinase activity, they play crucial roles as regulators of many cellular functions by mimicking classic kinases to mediate protein–protein interactions and downstream signaling transmission [1]. Peak1-related, kinase-activating pseudokinase 1 (PRAG1, molecular mass: 149.624 kDa), also known as pragmin or SGK223, belongs to the pseudopodium-enriched atypical kinase (PEAK) family of pseudokinase. It consists of an N-terminal domain (residues 1–216), an unstructured PEST linker (residues 217–931), and a C-terminal pseudokinase domain flanked by helical domains (residue 932–1406) [2]. PRAG1 has been reported to be highly expressed in various tissues, including the cerebral cortex of the brain, the respiratory system, and the gastrointestinal tract [3]. PRAG1 is located in the cytoplasm, cell junctions, focal adhesions, and the nucleus. The Rho family small GTPases, including Rho (A, B, C), Rac (1, 2, 3), Cdc42, and Rnd (1, 2, 3) have been reported to be involved in the regulation of the cytoskeleton, leading to subsequent morphological changes in a variety of cell types [4,5]. Early studies identified PRAG1 as an effector of Rnd2 that stimulates RhoA activity, thereby regulating neurite outgrowth [3]. Structural analysis revealed that PRAG1 can form homo-oligomers as well as hetero-oligomers with another pseudokinase, PEAK1 (also known as SGK269). These dimerization events are mediated by its pseudokinase domain, which stimulates C-terminal Src kinase (CSK) activity [6]. Recent studies have also revealed that PRAG1 colocalizes with CSK at focal adhesions to regulate cell morphology and motility [7]. Furthermore, overexpression of PRAG1 alone is sufficient to induce a more elongated, fibroblastic morphology [3]. The pseudokinase domain of PRAG1, located at its C-terminus, has been shown to be essential for its activity [8]. Therefore, PRAG1 serves as a crucial regulator of cell morphology, migration, and cancer cell invasion [9]. However, whether PRAG1 participates in the regulation of cell morphology under stress conditions remains unknown.

Remarkably, PRAG1 is largely unstructured [8]. Moreover, previous studies have reported that overexpression of PRAG1 forms punctate structures in cells [3], suggesting that PRAG1 may form functionally relevant phase-separated condensates.

In this study, we demonstrate that PRAG1 undergoes condensation, a process conserved across rodent and human cells. Further studies reveal that the formation of PRAG1 condensates depends on intermolecular interactions between its α-N and α-J helices. Additionally, TurboID-based proteomic analysis of PRAG1 condensates supports its role in modulating cell morphology and cell contraction. Notably, PRAG1’s function in mediating cell contraction is dependent on condensation. Furthermore, we observed a consistent increase in PRAG1 condensates under various stress conditions, suggesting that aberrant PRAG1 phase separation may contribute to various human disease.

## 2. Materials and Methods

### 2.1. Antibodies

The following antibodies were used in this study: PRAG1 (HPA012066, IF: 1:200, WB: 1:1000; MilliporeSigma, Burlington, MA, USA), Tyrosine Hydroxylase (45648, IF: 1:2000; Cell Signaling Technology (CST) Inc., Danvers, MA, USA), G3BP1 (13057-2-AP, IF: 1:200, WB: 1:1000; Proteintech Group Inc., Chicago, IL, USA), STREP II (AE066, IF: 1:500; ABclonal Technology, Woburn, MA, USA), Goat anti-Mouse IgG (H + L) Cross-Adsorbed Secondary Antibody, Alexa Fluor™ 594 (A-11005, IF: 1:1000; Thermo Fisher Scientific Inc., Waltham, MA, USA), Goat anti-Rabbit IgG (H + L) Cross-Adsorbed Secondary Antibody, Alexa Fluor™ 594 (A-11012, IF:1:1000; Thermo Fisher Scientific Inc., Waltham, MA, USA), Goat anti-Rabbit IgG (H + L) Cross-Adsorbed Secondary Antibody, Alexa Fluor™ 488 (A-11008, IF:1:1000; Thermo Fisher Scientific Inc., Waltham, MA, USA), Goat anti-Mouse IgG (H + L) Highly Cross-Adsorbed Secondary Antibody, Alexa Fluor™ 488 (A-11029, IF: 1:1000; Thermo Fisher Scientific Inc., Waltham, MA, USA), Horseradish Peroxidase-conjugated AffiniPure Goat Anti-Rabbit IgG (A0208, WB: 1:5000; Beyotime Biotechnology, Shanghai, China), Horseradish Peroxidase-conjugated AffiniPure Goat Anti-Mouse IgG (A0216, WB: 1:5000; Beyotime Biotechnology, Shanghai, China).

### 2.2. Chemicals

Rotenone (HY-B1756; MedChemExpress (MCE), Monmouth Junction, NJ, USA), carbonyl cyanide chlorophenylhydrazone (CCCP, HY-100941; MCE, Monmouth Junction, NJ, USA), Antifade Mounting Medium for fluorescence (with 4′,6-diamidino-2-phenylindole (DAPI)) (BL739A; Biosharp, Hefei, China), 1-methy-4-phenylpyridinium (MPP^+^) iodide (D048-100MG; MCE, Monmouth Junction, NJ, USA), Hydrogen peroxide solution (88597; MilliporeSigma, Burlington, MA, USA), Sodium arsenite (S7400-100G; MCE, Monmouth Junction, NJ, USA), Sorbitol (S1876-100G; MCE, Monmouth Junction, NJ, USA), Y-27632 (HY-10071; MCE, Monmouth Junction, NJ, USA), Cytochalasin D (N6682; MCE, Monmouth Junction, NJ, USA), Actin-Tracker Green-488 (C2201S; Beyotime Biotechnology, Shanghai, China), Actin-Tracker Red 555 (C2203S; Beyotime Biotechnology, Shanghai, China), 2× Phanta Max Master Mix (P515-01; Vazyme, Nanjing, China), protease inhibitor cocktail (P8340; MCE, Monmouth Junction, NJ, USA), RIPA (strong) (P0013B; Beyotime BIotechnology, Shanghai, China), D-biotin (B4501; MCE, Monmouth Junction, NJ, USA), recombinant RNasin Ribonuclease inhibitor (N2511; Promega Corporation, Madison, WI, USA), Cell Counting Kit-8 (CCK8) (FD3788-500T; FUdebio-tech (FD), Hangzhou, China), ClonExpress MultiS One Step Cloning Kit (C113-01; Vazyme, Nanjing, China), QuickCut DpnI (1609; Takara Bio Inc., Kusatsu, Japan), BeyoMag Streptavidin Magnetic Beads (P2151-1mL; Beyotime Biotechnology, Shanghai, China), Lipofectamine 3000 (L3000075; Thermo Fisher Scientific Inc.., Waltham, MA, USA), Enhanced BCA Protein Assay (P0009; Beyotime Biotechnology).

### 2.3. Cell Culture

Human neuroblastoma SH-SY5Y cells were cultured in Dulbecco’s modified Eagle’s medium/F12 (DMEM/F-12 GlutMAXTM medium supplemented with 10% fetal bovine serum (FBS) and 1% penicillin/streptomycin (P/S)). Human embryonic kidney HEK293T cells were cultured in DMEM media supplemented with 10% FBS and 1% P/S. Cells were cultured in a humidified incubator at 37 °C with 5% CO_2_. For confocal microscopy imaging, cells were seeded in 2 cm or 4-well confocal dishes; for flow cytometry, cells were seeded in 6-well plates; for the CCK8 assay, cells were seeded in 96-well plates; and for TurboID assay, cells were seeded in 9 cm plates.

### 2.4. Plasmid Contruction

Oligonucleotides were synthesized by SUNYA Biotechnology (Hangzhou, China). The full-length coding sequence of PRAG1 was amplified by PCR from SH-SY5Y cells-derived cDNA library with a total RNA extraction kit and First-Strand cDNA Synthesis kit (TIANGEN Biotech Co., Ltd., Beijing, China). The 2× Panta max Master Mix was used to amplified PCR fragments. Primers were designed to amplify the linearized vector of pcDNA3.1-CMV-EGFP (pcDNA3.1-CMV-()-GGSGGGSGGGSG-EGFP for N-terminal insertion of PRAG1, pcDNA3.1-CMV-EGFP-() for C-terminal insertion of PRAG1, pcDNA3.1-CMV-() for STREP-PRAG1 insertion). Linearized vectors were digested with QuickCutTM DpnI. Then, the cDNA of PRAG1 was cloned into related linearized vectors using the ClonExpress MultiS One Step Cloning kit. The ligation products were transformed into DH5α cells (Tsingke Co., Ltd., Hangzhou, China), and positive clones were selected and amplified via Ampicillin resistance and verified by sequencing (SUNYA Biotechnology, China) to ensure the correct in-frame fusion and location of PRAG1 with GFP tags. Primers containing the Met-STREP coding sequence were designed to amplify the cDNA-encoding STREP-PRAG1 from the verified pcDNA3.1-CMV-EGFP-GGSGGGSGGGSG-PRAG1 construct. The amplified cDNA was then inserted into a linearized vector (pcDNA3.1-CMV for STREP-PRAG1 insertion) using standard cloning techniques. Successful insertion was confirmed as described above.

### 2.5. Transfection

Cells were cultured to the required confluency (~80%). For LipofectamineTM3000-mediated transfection, 1 μg of plasmid and 1 μL of P3000 reagent were mixed with 50 μL of opti-MEMTM, and the mixture was incubated at room temperature (rt) for 5 min. Then, 1 μL of Lipofectamine^TM^ 3000 was added to the mixture and incubated for 15 min. The final mixture was added to the cells (1 mL culture medium for 24-well plate) and incubated for 24 h.

### 2.6. Immunostaining and Confocal Microscopy Imaging

Briefly, the cells plated on confocal dishes were initially fixed in 4% paraformaldehyde (PFA) for 20 min at room temperature. After washing twice with phosphate-buffered saline (PBS), the cells were blocked with 4% bovine serum albumin (BSA) and 0.5% PBST (PBS supplemented with 0.5% Triton X-100) for one hour at room temperature. Subsequently, the cells were incubated overnight at 4 °C with the appropriate primary antibodies diluted in 1% BSA and 0.1% PBST at optimized concentrations. The following day, the cells were washed three times in PBS and then incubated with the corresponding secondary antibodies. After washing three-times with PBS, a mounting medium containing DAPI was added for imaging. Fluorescence images were captured using an Olympus FV1000 microscope (Olympus Corporation, Tokyo, Japan) with a 63× objective.

### 2.7. Drug Treatment

All drugs were prepared as high concentration as stock solutions. Rotenone was dissolved in dimethyl sulfoxide (DMSO) to prepare a 1 mM stock solution, and CCCP was dissolved in DMSO to prepare a 10 mM stock solution. D-sorbitol was dissolved in the culture medium to prepare a 0.4 M solution. MPP^+^ was dissolved in H_2_O to prepare a 10 mM stock solution. A 3% H_2_O_2_ solution was dissolved in the culture medium to prepare a 1 mM solution. For drug treatment, the cells were separately treated with 1 μM rotenone for 12 h, 10 μM CCCP for 30 min, 0.4 M D-sorbitol for 30 min, 100 μM MPP^+^ for 12 h, and 1 mM H_2_O_2_ for 30 min. For treatment with pre-formed fibrils (PFFs) of α-syn labeled with Cy5 (PFF-Cy5, kindly provided by Jingwei Zhao Lab, Zhejiang University, Hangzhou, China), the cells were treated with 10 μM PFF-cy5 for 12 h, and then, the cells were fixed and immunostained for imaging.

### 2.8. Cell Counting Kit-8 (CCK8) Assay

Cell viability was assessed using the Cell Counting Kit-8 (CCK8) assay according to the manufacturer’s instructions. Briefly, 100 μL of cell suspension was seeded into 96-well plates at a density of 10 × 10^3^ cells/well and allowed to adhere overnight. After treatment with drugs at the specified concentration for the indicated duration, 10 µL of CCK8 solution was added to each well, and the plates were incubated at 37 °C for 3 h. Absorbance was measured at 450 nm using a SpectraMax iD5 microplate reader (Molecular Devices, Sunnyvale, CA, USA). Results were normalized to untreated controls and expressed as percentage viability.

### 2.9. Western Blotting

Unless stated otherwise, all the following steps were performed at 4 °C on ice: After washing twice with PBS, the cells were collected and lysed with strong RIPA (Fudebiotech, Hangzhou, China) supplemented with 100× protease inhibitor for 20 min on a rotator incubator. Subsequently, after further lysis by sonication, the lysates were centrifuged for 30 min. The protein concentration of the supernatants was measured using the BCA assay. Then, after adjusting to a consistent concentration, the samples were boiled for 6 min and loaded on sodium dodecyl sulfate–polyacrylamide gel electrophoresis (SDS-PAGE) gels for protein separation. Subsequently, after transferring (by electroblotting) the separated proteins onto a polyvinylidene fluoride membrane, the target proteins were identified by immunoreacting with specific primary and corresponding secondary antibodies. Finally, following imaging and densitometric analysis of immunoreactive protein bands, and the band intensities were quantitatively analyzed using open-source image processing Fiji 2.16.0 software package (https://imagej.net/software/fiji/, accessed on 27 September 2022). Original figures can be found in Supplementary Materials.

### 2.10. FRAP

Fluorescence recovery after photobleaching (FRAP) experiments were performed using an Olympus FV1000 confocal microscope equipped with a 60× oil immersion objective. SH-SY5Y cells were plated onto confocal dishes and transfected with desired fluorescent-tagged plasmids. Following a 24 h incubation period, droplets were photobleached using a laser intensity of 100% at 488 nm (for GFP) for 2 s, and recovery was monitored every 1 s for 2 min and 30 s. The fluorescence intensity prior to bleaching was normalized to 1, and the signal post-bleaching was normalized relative to the pre-bleached level. The relative intensity was calculated using the formula:

(Fpost − Fbackground)/(Fpre − Fbackground).

### 2.11. Light-Induced Condensation

SH-SY5Y cells were seeded onto confocal dishes and transfected with CMV-CRY2-mCherry, CMV-CRY2-mCherry-PRAG1 plasmids for 24 h. Subsequently, images were captured using an Olympus FV1000 confocal microscope equipped with a 60× oil immersion objective. Cells expressing mCherry were subjected to stimulation with a 488 nm laser for 2 s at 5% intensity. Images were captured every 1 s.

### 2.12. TurboID Assay

HEK293T cells were seeded in 10 cm culture dishes and cultured to 80% confluency. Subsequently, the cells were transfected with plasmids encoding GFP-TurboID and GFP-PRAG1-TurboID. After a 48 h incubation period, biotin was added into the medium, adjusting the concentration to 500 µM, and the cells were further incubated for 15 min at 37 °C. Biotin labeling was terminated by transferring the cells to 4 °C, followed by three washes with cold PBS. After centrifugation at 1000× *g* for 3 min, the cell pellet was lysed with a weak RIPA buffer containing a protease inhibitor cocktail and an RNase inhibitor for 20 min. After centrifuging the lysates at 20,000× *g* and 4 °C for 30 min, each supernatant was collected and combined with 100 µL streptavidin magnetic beads. The mixture was incubated overnight at 4 °C with gentle rotation. Subsequently, the supernatant was discarded, and the beads were washed sequentially, twice with 1 mL of weak RIPA buffer, once with 1 mL of 1 M KCl, once with 1 mL of 0.1 M Na_2_CO_3_, once with 1 mL of 2 M urea in 10 mM Tris-HCl (pH 8.0), and twice with 1 mL of RIPA (weak) buffer. Biotinylated proteins were then eluted using 2× SDS loading buffer. The resulting supernatant was loaded on an SDS-PAGE gel, and protein bands of interest, as indicated by their molecular weight, were collected for subsequent mass spectrometry (MS) analysis (Applied Protein Technology, Co., Ltd., Shanghai, China). The MS data were obtained from four biological replicates, analyzed using the MaxQuant software version 1.6.14.0 (Max Planck Institute of Biochemistry, Martinsried, Germany). Proteins specifically detected in GFP-PRAG1-TurboID but not in GFP-TurboID were subjected to Kyoto Encyclopedia of Genes and Genome (KEGG) functional enrichment analysis using KOBAS 3.0 (http://bioinfo.org/kobas, accessed on 30 November 2022).

### 2.13. Generation of Knock-Out Cell Line

The knock-out cell lines were generated using the pX300 plasmid system (#110403, Addgene, Cambridge, MA, USA). In summary, oligonucleotides encoding guide RNAs targeting the N terminus of PRAG1 were inserted into a Bbs I-cleaved pX330 backbone. The sequence targeted for PRAG1 was 5′-GCGGAGCGACCACCAGCTGG-3′. Plasmids were verified for sequence accuracy and co-transfected with a plasmid harboring a puromycin resistance gene using lipofectamine 3000. After a 24 h incubation period, cells were subjected to selection with 3 μg/mL puromycin. After 7 days of selection, cells were individually sorted by flow cytometry cell sorting using a Beckman Coulter MoFlo Astrios EQ cell sorter (Beckman Coulter Inc., Brea, CA, USA) and seeded into 96-well plates. Knock-out single-cell clones were subsequently validated by DNA sequencing and Western blot analysis.

### 2.14. Software and Database

Software used in this study: Fiji (https://imagej.net/software/fiji/, accessed on 27 September 2022; used for image processing), GraphPad Prism 9 (used for statistical analysis; GraphPad Software Inc., San Diego, CA, USA), KOBAS 3.0 (http://bioinfo.org/kobas, accessed on 30 November 2022; used for Kyoto Encyclopedia of Genes and Genome (KEGG) pathway enrichment analysis), MEGA11 (https://www.megasoftware.net/dload_win_beta, accessed on 23 August 2024 used for sequence alignment). MaxQuant software version 1.6.14.0 (Max Plank Institute of Biochemistry, Martinsried, Germany, used for raw mass-spectrometry data processing).

Databases resources: Human Protein Atlas (https://www.proteinatlas.org/, accessed on 1 August 2023, used for expression profile of human PRAG1), Uniprot (https://www.uniprot.org/, accessed on 1 August 2023, used for protein sequence retrieval and sequence alignment), National Center for Biotechnology Information (NCBI, Bethesda, MD, USA; (https://www.ncbi.nlm.nih.gov/; accessed on 5 April 2023, used for coding sequence retrieval), Single-cell genomic profiling of human dopamine neurons (https://singlecell.broadinstitute.org/single_cell/study/SCP1768/single-cell-genomic-profiling-of-human-dopamine-neurons-identifies-a-population-that-selectively-degenerates-in-parkinsons-disease-single-nuclei-data, accessed on 27 September 2023).

### 2.15. Statistical Analysis

The data were analyzed using GraphPad Prism 9 software (GraphPad Software Inc., San Diego, CA, USA) and presented as the mean ± sem otherwise specified. All experiments were repeated in at least three independent biological replicates. The normality of quantitative data distributions was assessed using the Shapiro–Wilk’s test. For comparison of multiple groups, a one-way ANOVA with Dunnett’s multiple comparisons test was used to compare the means of three or more independent groups, and a two-way ANOVA with Sidak’s multiple comparisons test was used to analyze the effects of two categorical independent variables. A two-tailed Student’s *t*-test was used to identify significant individual differences between two groups, using the Mann–Whitney test used in cases where the data did not adhere to a normal distribution. Statistical significance was defined as *p* <  0.05 in all analyses.

## 3. Results

### 3.1. PRAG1 Forms Dynamic Condensates in Cells

**Figure 1:**
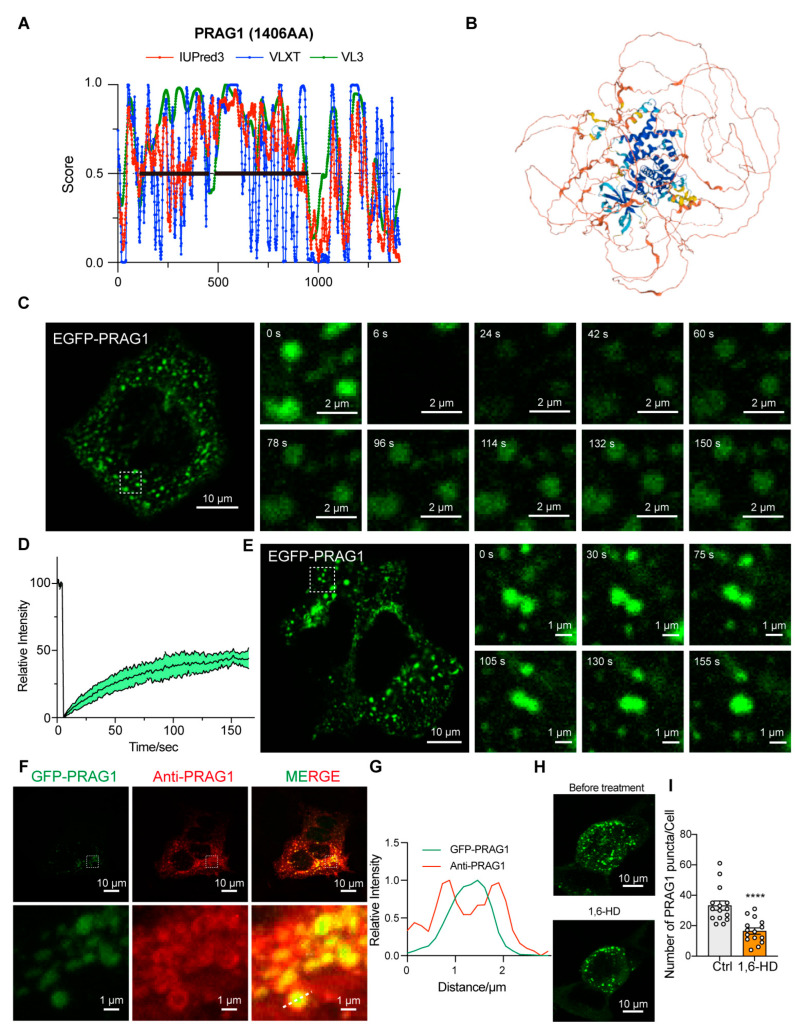
PRAG1 forms condensates in cells. (**A**) Protein sequence of PRAG1 was analyzed with IUPred 3, PONDR-VLXT, PONDR-VL3 algorithms. The IDRs regions are annotated with bold lines. (**B**) Predicated structure of PRAG1 using the AlphaFold2 artificial intelligence system. (**C**) Confocal images of representative SH-SY5Y cells expressing the EGFP-PRAG1 construct. EGFP-PRAG1 protein was diffusely distributed throughout the cell body with punctate structures and aggregates with irregular shape. Scale bar: 10 μm. Fluorescence recovery after photobleaching (FRAP) analysis showed the droplet-like characteristic of EGFP-PRAG1 puncta. Scale bar: 2 μm. (**D**) FRAP analysis of EGFP-PRAG1 in transfected SH-SY5Y cells. Five condensates from five different cells were bleached and imaged. Data represents mean ± sem. (**E**) Live-cell images showing the fusion of EGFP-PRAG1 condensates into larger condensates in transfected SH-SY5Y cells. Scale bar, 10 μm. (**F**) SH-SY5Y cells expressing EGFP-PRAG1 were stained with anti- PRAG1antibody. Scale bar, 10 μm. (**G**) Line scans show the related intensity profiles of GFP-PRAG1 and Anti-PRAG1. (**H**) Representative images showing GFP-PRAG1 condensates were disrupted by 1.5% 1,6-hexanediol (1,6-HD) treatment for 5 min. (**I**) Quantification of the number of EGFP-PRAG1 condensates after 1,6-HD treatment. *n* = 15. Students’ *t* test. **** *p* < 0.0001.

PRAG1, a member of the PEAK family of pseudokinase, was found to be widely expressed in various human tissues, including brain, respiratory system, gastrointestinal tract, and kidneys (Figure S1). Notably, PRAG1 was found to be highly expressed in the brain, particularly in excitatory neurons (Figure S1). PONDR (VLXT, VL3-BA) and IUPred3 are widely utilized in recent studies for predicting protein disorders. These tools provide a disorder score for each residue, with regions scoring above 0.5 typically being classified as intrinsically disordered [10,11,12,13,14]. A sequence analysis of human PRAG1, using the IUPred 3 and PONDR prediction algorithms, predicted that the N-terminal region of PRAG1 is intrinsically disordered (Figure 1A). Although previous studies have reported the crystal structure of the C-terminus of PRAG1, the N-terminus appears to be disordered, as suggested by the predicted structural model (Figure 1B, the area highlighted in light color). Given that previous studies have reported that PRAG1 forms punctate structures [3], we sought to further investigate whether PRAG1 forms dynamic condensates in cells by exogenously overexpressing PRAG1 in human neuroblastoma SH-SY5Y cells. Exogenously expressed PRAG1 exhibited a diffuse distribution throughout the cell body, with the presence of some spherical structures and irregular-shaped aggregates (Figure 1C). the FRAP analysis of spherical GFP-PRAG1 puncta showed 44.54 ± 4.55% fluorescence recovery after 2 min 30 s, indicating their dynamic properties and 44.54% of mobile fractions (Figure 1C,D). Live imaging analysis revealed that PRAG1 puncta undergo fusion events (Figure 1E). These findings suggest that PRAG1 condensates exhibit dynamic properties and engage in molecular exchange with the surrounding environment.

To eliminate the potential biases arising from the position, type, or size of the tag, we generated a PRAG1-EGFP construct. Expression of this construct in SH-SY5Y cells resulted in punctate structures that exhibited fluorescence recovery after photobleaching (Figure S2A,B). Additionally, expression of the mCherry-PRAG1 construct led to the formation of condensates that colocalized with EGFP-PRAG1 condensates (Figure S2C), indicating that the type of fluorescence tag does not influence the formation of PRAG1 condensates. Immunostaining of exogenously overexpressed PRAG1 with a PRAG1 antibody showed that both EGFP-PRAG1 and PRAG1-EGFP condensates were surrounded by the PRAG1 antibody signal (Figure 1F,G and Figure S2D,E). In addition, STREP-PRAG1 construct, which contains the smaller STREP tag (~2 kDa, compared to 27 kDa for EGFP), also formed condensates in SH-SY5Y cells that were detectable by the PRAG1 antibody (Figure S2F,G). These results demonstrate that the tag size does not affect PRAG1 condensates formation. Although EGFP-PRAG1 formed protein aggregates, which may result from its natural ability to undergo oligomerization [8], PRAG1-EGFP condensates showed only 18.21 ± 6.20% fluorescence recovery after 2 min 30 s, indicating that the majority of PRAG1-EGFP forms immobile aggregates (Figure S2A,B). Additionally, we observed that treatment with 1.5% 1,6-hexanediol, a reagent commonly used to dissolve condensates formed via phase separation [15,16], led to partial disruption of EGFP-PRAG1 condensates (Figure 1H,I). These liquid-like dynamic properties suggest that PRAG1 condensates are likely formed through liquid–liquid phase separation. (LLPS). Therefore, we opted to label PRAG1 with GFP at its N-terminus for subsequent experiments.

### 3.2. PRAG1 Is Conserved in Human and Rodents

**Figure 2:**
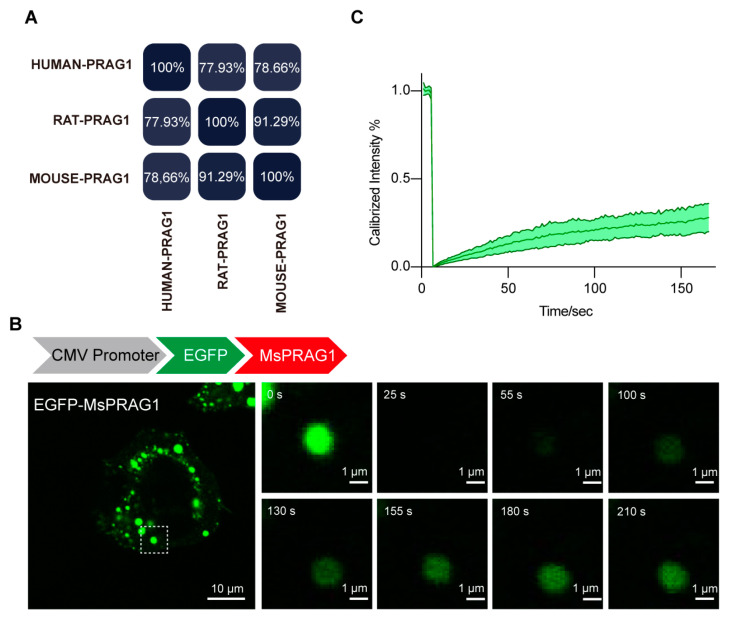
PRAG1 is conserved in humans and rodents. (**A**) Percent identity matrix depicting the conservation of PRAG1. (**B**) Confocal images of representative SH-SY5Y cells expressing EGFP-mouse PRAG1. The EGFP-mouse PRAG1 protein was diffusely distributed throughout the cell body with punctate structures. Scale bar: 10 μm. Fluorescence recovery after photobleaching (FRAP) analysis showed the dynamic property of EGFP-PRAG1 puncta. (**C**) FRAP analysis of mouse PRAG1. Ten condensates from four different cells were bleached and imaged.

To verify the evolutionary conservation of PRAG1 condensation, we performed homology analysis of the amino acid (AA) sequence of PRAG1 proteins from *Homo sapiens, Mus musculus*, and *Rattus norvegicus*. Compared with the human PRAG1 sequence, mouse and rat PRAG1 exhibited 78.66% and 77.93% homology, respectively (Figure 2A and Figure S3), and the homology between mouse PRAG1 and rat PRAG1 was 91.29% (Figure 2A and Figure S3). Additionally, we confirmed that EGFP-mouse PRAG1 forms condensates with dynamic properties, as the fluorescence of mouse PRAG1 condensates also recovered after photobleaching (Figure 2B,C). However, FRAP analysis of spherical GFP-mouse PRAG1 puncta showed 25.88 ± 8.21% fluorescence recovery after 2 min 30 s, indicating the majority of immobile fraction of EGFP-mouse PRAG1 when expressed in human SH-SY5Y cells. These findings, to some extent, suggest that the formation of PRAG1 condensates is conserved across humans and rodents, although the dynamic properties show some variability.

### 3.3. Both IDRs and Structured Regions Are Required for PRAG1 Condensation

**Figure 3:**
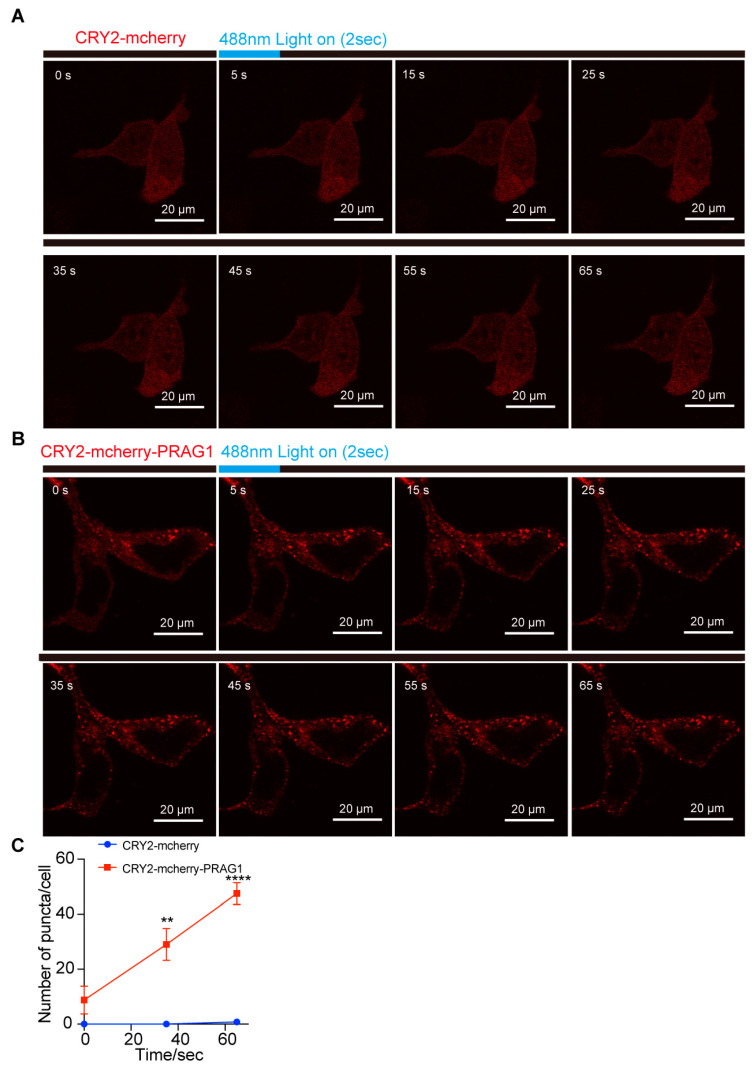
PRAG1 forms condensates in cells. (**A**,**B**) SH-SY5Y cells were transfected with CRY2-mCherry and CRY2-mCherry-PRAG1 plasmids for 24 h, respectively. Then, the cells with mCherry fluorescence were exposed to 488 nm laser light (Intensity 5% for 2 s). Confocal time-lapse images showing the appearance of light-induced puncta in CRY2-mCherry-PRAG expressing cells within 3 s after blue light exposure. (**C**) The number of puncta in cells expressing CRY2-mCherry or CRY2-mCherry-PRAG1 was quantified over time. *n* = 4. ** *p* < 0.01. **** *p* < 0.001. Students’ *t* test.

Due to the instability of purified PRAG1 and its propensity to aggregate, we were unable to perform an in vitro LLPS assay to generate a phase diagram for PRAG1. To further investigate its potential for phase separation, we generated a CRY2-mCherry-PRAG1 construct (optoPRAG1) for subsequent experiments. CRY2-mCherry exhibited a diffused distribution, and its self-association is initiated upon exposure to 488 nm blue light but did not form condensates in the cells (Figure 3A,C) [17]. Prior to blue light exposure, we observed that, at low expression levels, optoPRAG1 showed a diffuse distribution throughout the cell body, whereas cells with high expression level of optoPRAG1 showed pre-existing condensates (Figure 3B,C and Figure S4A). These condensates showed comparable size to Mcherry-PRAG1-formed condensates while lacking the ability of undergo FRAP (Figure S4A,B). Time-lapse imaging revealed the gradual formation of PRAG1 condensates in the cytoplasm of low optoPRAG1-expressing cells upon blue light stimulation (Figure 3B,C), and the cells with pre-existing condensates also showed increased PRAG1 condensates (Figure 3B,C), but this phenomenon did not occur in the CRY2-mCherry transfected cells (Figure 3A,C). Furthermore, we found the formation of optoPRAG1 condensates is dependent on both the expression level and the duration of light exposure (Figure S4C,D). These findings provide additional evidence supporting the potential of PRAG1 to undergo phase separation. However, the light-induced condensates exhibited limited solubility and displayed deficient FRAP (Figure S4E–G), suggesting that the Cry2 tag negatively affect the dynamic properties of Mcherry-PRAG1 condensates.

**Figure 4:**
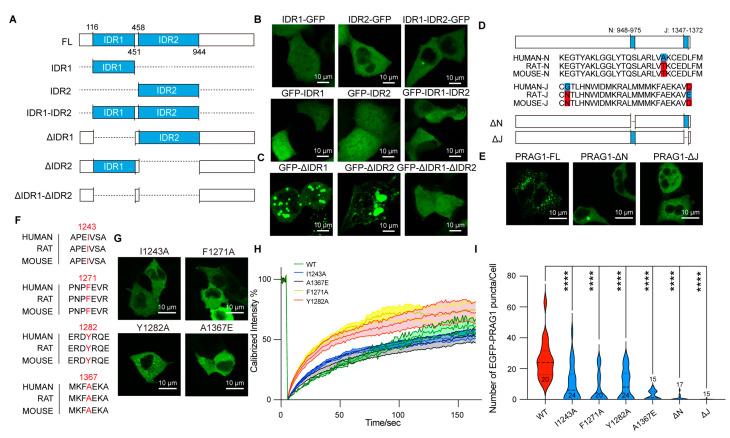
PRAG1 forms condensates in cells via its N and J helix domains. (**A**) Scheme showing the IDRs of PRAG1 tagged with EGFP at the N or C terminus. (**B**,**C**) Representative confocal images showing the distribution of the recombinant GFP-labeled IDRs (**B**) and IDR-deletion mutants (**C**) in SH-SY5Y cells. (**D**) Scheme showing the EGFP-PRAG1 with N-helix or J-helix domain deletion. (**E**) Representative confocal microscopy images showing the distribution of the recombinant proteins in SH-SY5Y cells. Scale bar, 10 μm. (**F**) Sequence alignment showing the conservation of I1243, F1271, Y1282, and A1367 in human, rat, and mouse. (**G**) Confocal images showing the cell morphology of SH-SY5Y cells transfected with EGFP-PRAG1 mutants; then, the cells were stained with actin-tracker 555 and DAPI. WT: 12 puncta, 6 cells, I1243A: 12 puncta, 8 cells, F1271A: 9 puncta, 9 cells, Y1282A: 4 puncta, 4 cells, A1367E: 6 puncta, 4 cells. (**H**) FRAP analysis of EGFP-PRAG1 mutants. (**I**) Quantification of the number of PRAG1 puncta in cells expressing PRAG1 mutants. **** *p* < 0.0001; one-way ANOVA with Dunnett’s multiple comparisons test.

Based on the combined predicted disorderliness scores of the PRAG1 sequence obtained by the PONDR (VLXT, VL3-BA) and IUPred 3 algorithms, we annotated the regions AA116-AA451 and AA458-944 as intrinsically disordered regions (IDRs) (Figure 1A). The expression of EGFP-IDR1, EGFP-IDR2, and EGFP-IDR1-IDR2 did not lead to the formation of spherical structures in the cytosol of SH-SY5Y cells, nor did the expression of the C-terminally labeled IDRs (Figure 4A–C), suggesting that the IDRs alone are not sufficient for condensation. Additionally, the deletion of IDR1 resulted in the formation of larger puncta and fewer irregular aggregates (Figure 4D), which differed from those formed by full-length PRAG1. In contrast, the deletion of IDR2 resulted in the formation of fibril-like structures and giant irregular assemblies (Figure 4C). The deletion of both IDR1 and IDR2 completely abolished PRAG1 condensation (Figure 4D). These results suggest that the IDRs of PRAG1 are required for condensation.

Previous studies have shown that the N-helix (AA948-975) and J-helix (AA1347-1372) domains at the PRAG1 C-terminus mediate its dimerization [6]. To determine whether the N-helix or J-helix influences PRAG1 condensate formation, we generated EGFP-tagged PRAG1-ΔN and PRAG-ΔJ deletion constructs (Figure 4D,E). Expression of either EGFP-PRAG1-ΔN or EGFP-PRAG-ΔJ failed to produce condensates in SH-SY5Y cells (Figure 4E). These results indicate that the N and J helixes domains are required for PRAG1 condensation.

We also sought to identify single-site mutations that disrupt condensation while preserving the sequence integrity of PRAG1. The αG helix/A-loop interface of PRAG1 has been reported to be indispensable for homo-oligomerization. The size-exclusion chromatography analysis showed that F1271A, Y1282A, and I1243A mutants showed a reduction in higher order oligomers compared to the wild-type PRAG1 [9]. Additionally, Lecointre et al. demonstrated that the A1367E mutation in the C-terminus of PRAG1 destabilizes its dimerization [6]. We confirmed, by sequence alignment analysis, the high conservation of these amino acid residues (Figure 4F). To evaluate the effect of these mutants on PRAG1 condensation, we generated EGFP-tagged PRAG1 mutants and transfected them into SH-SY5Y cells (Figure 4G). FRAP analysis revealed that I1243A and F1271A mutations did not affect the dynamics of PRAG1 condensates, while The Y1282A and A1367E mutants exhibited a faster fluorescence recovery, reaching 50% of the fluorescence intensity more rapidly (T_1/2,I1243A_: 129.5 s, T_1/2,F1271A_: 146.1 s, T_1/2,Y1282A_: 46.5 s, T_1/2,A1367E_: 37.6 s, T_1/2,WT_: 105.1 s) (Figure 4H). Confocal imaging showed a significant reduction in the number of condensates in cells expressing PRAG1 mutants compared to those expressing wild-type PRAG1 (Figure 4I), indicating the impaired condensation ability in PRAG1 mutants.

### 3.4. PRAG1 Condensation Mediates Cell Contraction

**Figure 5:**
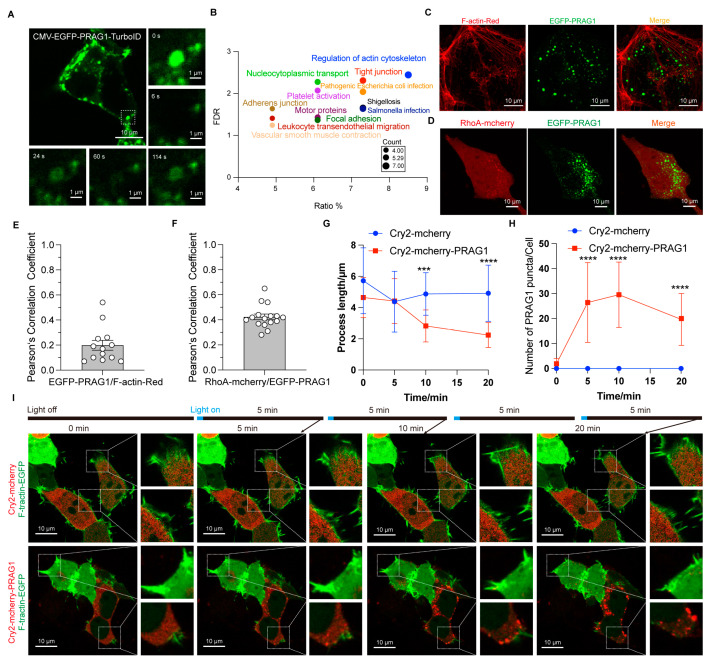
PRAG1 condensation function in promoting cell contraction. (**A**) Confocal images showing that the EGFP-PRAG1-turboID construct does not influence the formation of condensates. (**B**) Bubble map of the KEGG pathway enrichment analysis results of the PRAG1 interactome. (**C**) Representative confocal images showing the colocalization between EGFP-PRAG1 and F-tractin-EGFP in SH-SY5Y cells. (**D**) Representative confocal images showing co-localization of PRAG1 condensates and RhoA-mCherry in SH-SY5Y cells. (**E**) Pearson’s correlation coefficient analysis of F-actin with GFP-PRAG1 in panel (**C**). (**F**) Pearson’s correlation coefficient analysis of RhoA-mCherry with GFP-PRAG1 in panel (**D**). (**G**) Quantification of the process length shown in panel (**I**). One-way ANOVA with Dunnett’s multiple comparison’s test. Data are presented as the mean ± SD. *n* ≥ 15. *** *p* < 0.001, **** *p* < 0.0001. (**H**) Quantification of the number of PRAG1 puncta shown in panel (**I**). One-way ANOVA with Dunnett’s multiple comparison’s test. Data are presented as the mean ± SD. *n* ≥ 15. **** *p* < 0.0001. (**I**) SH-SY5Y cells were transfected with CRY2-mCherry-PRAG1/F-tractin-EGFP or CRY2-mCherry/F-tractin-EGFP constructs. Then, the cells were stimulated with a 488 nm laser light for 20 min. Since F-tractin-EGFP were imaged using a 488 nm laser light with higher power, and optoPRAG1 condensation also requires a 488 nm laser, to minimize potential cellular damage caused by long-time exposure to laser light, the cells were imaged for 1 s every 5 min.

The TurboID-mediated proximity biotinylation assay enables rapid tagging of close protein interactions and has been used to investigate the proteomics of functional condensates [10,18]. Thus, to investigate the function of PRAG1 condensates, we transfected GFP-TurboID and GFP-PRAG1-TurboID constructs into HEK293T cells to examine the PRAG1 condensate-specific interactome. As shown in Figure 5A, the GFP-PRAG1-TurboID construct retained its ability to form condensates, and the FRAP analysis confirmed its dynamic properties. Additionally, gene functional classification revealed that proteins enriched in PRAG1-TurboID-mediated biotinylated proteome are involved in the regulation of the actin cytoskeleton, cell junction, cell contraction, and cell adhesion (Figure 5B). To confirm these findings, we examined the colocalization of PRAG1 condensates with proteins with high enrichment scores. The results showed that some PRAG1 droplets were found in close proximity to F-actin, partially overlapping but not occupying the exact same space (Figure 5C,E), and PRAG1 condensates also colocalized with RhoA (Figure 5D,F), a protein known to promote cell contraction [3]. To further confirm the role of PRAG1 condensation in the regulation of cell contraction, we transfected CRY2-mCherry-PRAG1 and F-tractin-EGFP constructs into SH-SY5Y cells, which were then stimulated with a 488 nm laser light. We observed that blue-light-induced PRAG1 condensation led to decreased process length and F-actin disassembly, indicating the occurrence of process retraction and cell contraction (Figure 5G–I). However, this phenomenon was not observed in cells transfected with CRY2-mCherry and F-tractin-EGFP constructs (Figure 5G–I). Together, these results clearly demonstrate that PRAG1 condensation mediates cell contraction.

### 3.5. Stress Induces PRAG1 Puncta Formation

**Figure 6:**
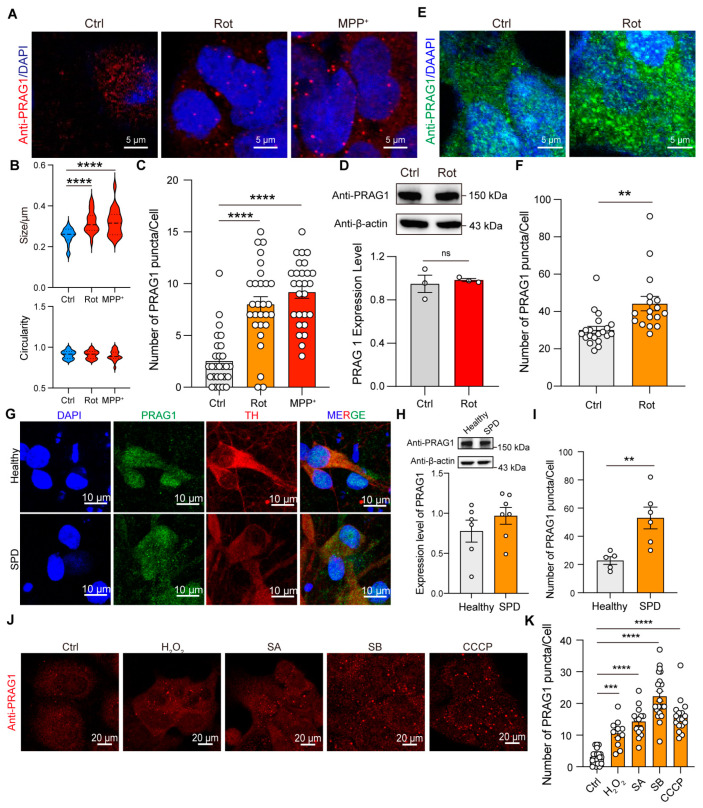
Stress induces the formation of PRAG1 puncta. (**A**) Confocal images of endogenous PRAG1 puncta stained with anti-PRAG1 antibody in SH-SY5Y cells with control (DMSO, 1 μL/mL), rotenone-conditioned (1 μM) or MPP^+^ (10 μM) medium for 12 h. (**B**) Plots showing the size and circularity of anti-PRAG1 signal. One-way ANOVA with Dunnett’s multiple comparisons test. *n* = 20. **** *p* < 0.0001. (**C**) Quantification of the number of PRAG1 puncta per cell in (**A**). One-way ANOVA with Dunnett’s multiple comparisons test. *n* = 28, 27, 27, respectively. **** *p* < 0.0001. (**D**) Western blot analysis of the expression level of endogenous PRAG1 in SH-SY5Y cells with control (Ctrl, 1 μL/mL), rotenone-conditioned (1 μM) medium. Students’ *t* test. ns, non-significant. (**E**) Confocal images of endogenous PRAG1 puncta stained with anti-PRAG1 antibody in HEK293T cells with control (Ctrl, 1 μL/mL), rotenone-conditioned (1 μM) medium for 12 h. (**F**) Quantification of the number of PRAG1 puncta per cell in (**E**). *n* = 20, 17. Students’ *t* test. ** *p* < 0.001. (**G**) Confocal images showing the expression pattern of PRAG1 in iPSC-differentiated DA neurons. TH, tyrosine hydroxylase. (**H**) Expression level of PRAG1 in iPSC-differentiated DA neurons is determined using Western blot analysis; down, quantification of the PRAG1 level. Students’ *t* test. (**I**) Quantification of the number of PRAG1 puncta per cell in (**G**). Students’ *t* test. ** *p* < 0.01. (**J**) Representative confocal images showing the effect of 1 mM hydrogen peroxide (H_2_O_2_), 0.4 M D-sorbitol (SB), 0.5 μM sodium arsenite (SA), or 10 μM CCCP treatment for 30 min on PRAG1 condensates in SH-SY5Y cells. (**K**) Quantification of the number of PRAG1 puncta per cell in (**J**). One-way ANOVA with Dunnett’s multiple comparisons test. *n* = 21, 11, 14, 21, 27, respectively. *** *p* < 0.001, **** *p* < 0.0001.

To determine whether PRAG1 forms condensates at endogenous levels, we performed immunostaining for PRAG1 using a primary antibody against PRAG1. PRAG1 showed a diffused distribution with several spherical puncta at normal conditions, similar to the condensates formed by EGFP-PRAG1 (Figure 6A), indicating its ability to form condensates. To determine whether PRAG1 plays a role in the cellular stress response, we treated SH-SY5Y cells with various stress inducers. First, we examined endogenous PRAG1 following rotenone treatment, a mitochondrial complex I inhibitor commonly used in Parkinson’s disease (PD) models [19]. As shown in Figure 6A, rotenone treatment resulted in a notable increase in the number and size of spherical PRAG1 puncta in SH-SY5Y cells (Figure 6A–C), while the expression level of PRAG1 remained unchanged (Figure 6D). This suggests that the formation of PRAG1 puncta is independent of expression changes. Similarly, treatment with MPP^+^, another mitochondrial complex I inhibitor [20], resulted in a comparable increase in PRAG1 puncta (Figure 6A–C). Additionally, we observed that the size of EGFP-PRAG1 condensates increased, while their number remained unchanged following treatment with rotenone or MPP^+^ (Figure S5A–C). These findings indicate that the increase in PRAG1 puncta is not specific to rotenone treatment but rather represents a general response to mitochondrial stress. To determine whether this effect is cell-type-specific, we performed immunostaining of PRAG1 in rotenone-treated HEK293T cells. Confocal imaging also revealed an increase in PRAG1 puncta in HEK293T cells (Figure 6E,F). Additionally, our recent study identified mitochondrial dysfunction in induced potential stem cell (iPSC)-derived DA neurons from a PD patient compared to those derived from a healthy individual [21]. Immunostaining for PRAG1 in differentiated DA neurons from both healthy and PD groups demonstrated that the PD group showed a significantly higher number of PRAG1 puncta while PRAG1 expression levels were unaffected (Figure 6G–I). These results confirm that mitochondrial stress promotes the formation of PRAG1 puncta. To determine whether PRAG1 responds differently to various stress conditions, we exposed SH-SY5Y cells to H_2_O_2_ (oxidative stress) [22], D-sorbitol (SB, osmotic stress) [13], sodium arsenite (SA, oxidative stress) [23], and carbonyl cyanide 3-chlorophenylhydrazone (CCCP, mitochondrial potential inhibitor) [24]. Similar to the effects observed with rotenone and MPP^+^, treatment with H_2_O_2_, SB, and SA also led to an increase in PRAG1 puncta (Figure 6J,K). Collectively, these findings suggest that the PRAG1 puncta formation represents a general mechanism activated in response to diverse cellular stressors.

PRAG1 puncta increased following treatment with rotenone and MPP^+^, both commonly used in PD models, suggesting a potential link between PRAG1 and PD. The formation of Lewy bodies (LBs) and the degeneration of vulnerable dopamine (DA) neurons in the substantia nigra pars compacta (SNc) are hallmark features of PD, with the major component of LBs identified as α-synuclein (α-syn)-derived fibrils [21,25]. Previous studies have shown that α-syn undergoes LLPS to form condensates, which also respond to stress [26]. To determine whether PRAG1 puncta colocalize with α-syn-formed puncta, SH-SY5Y cells were treated with pre-formed fibrils (PFFs) of α-syn. Confocal imaging revealed that PRAG1 puncta do not colocalize with α-syn PFF puncta (Figure S5D,E), indicating that mitochondrial stress-induced PRAG1 puncta are not associated with α-syn aggregation.

Single-nucleus RNA sequencing (snRNA-seq) of dopaminergic (DA) neurons from postmortem human SNc have annotated that the SOX6-AGTR1 positive neurons are the most vulnerable neurons in the human SNc [27]. The G-protein-gated inwardly-rectifying K^+^ channel subunit 2 (GIRK2, encoded by the KCNJ6 gene) has been identified as a marker for vulnerable SNc DA neurons, while calbindin 1 (encoded by the CALB1 gene) is a marker for invulnerable DA neurons in mammals [28]. We found that the KCNJ6 gene is more highly expressed in the SOX6-AGTR positive neuron cluster (Figure S5F,G), whereas the CALB1 gene is not (Figure S5H). Remarkably, PRAG1 is more prominently enriched in the vulnerable neuron cluster, similar to KCNJ6 (Figure S5I).

Previous studies have identified PRAG1 as a component as a component of stress granules (SGs) based on mass spectrometry data [17]. Additionally, sodium arsenite has been reported to induce SG formation [13,29]. Thus, we examined the distribution of PRAG1 puncta and SGs under stress. In the absence of stress, PRAG1 puncta were present, but SGs were rarely detected (Figure S5J,K). However, sodium arsenite treatment triggered a robust SG formation and an increase in PRAG1 puncta (Figure S4C). Although these two condensates showed increased colocalization upon sodium arsenite treatment (Figure S5J,K), the majority of PRAG1 puncta did not overlap with SGs (Figure S4C,D). These results support the notion that PRAG1 forms SG-independent puncta and is closely associated with the cellular stress response. We also attempted to generate cell lines with in situ GFP-labeled PRAG1 to confirm the dynamic properties of these PRAG1 puncta at the endogenous level, but we failed due to technical limitations. Therefore, these endogenous PRAG1-formed spherical structures were temporarily termed with neutral puncta rather than dynamic condensates, as in the case of EGFP-PRAG1. However, the formation of stress-induced spherical puncta rather than irregular protein aggregates demonstrates, to some extent, the dynamic properties of PRAG1.

### 3.6. PRAG1-Mediated Cell Contraction Depends on Its Condensation Ability

**Figure 7:**
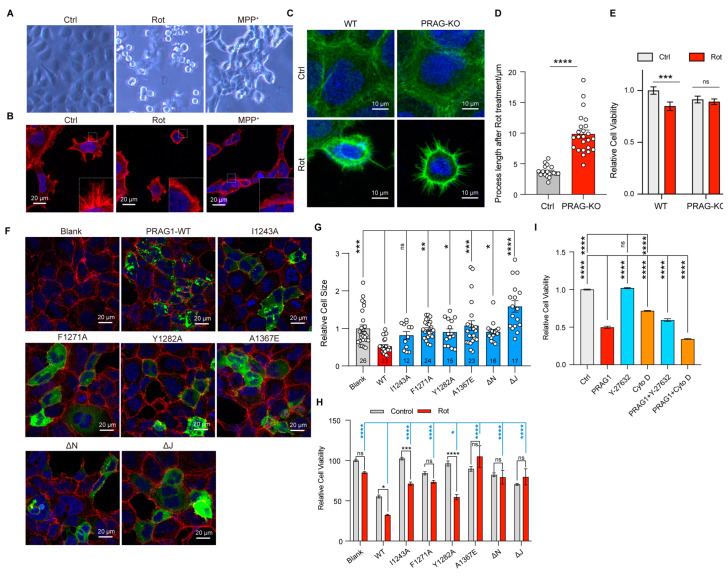
PRAG1-mediated cell contraction depends on its condensation ability. (**A**) DIC images showing the morphology of SH-SY cells with control (DMSO, 1 μL/mL), rotenone-conditioned (1 μM), or MPP^+^ (10 μM) medium for 12 h. (**B**) Representative confocal images showing the process of SH-SY5Y cells with control (DMSO, 1 μL/mL), rotenone-conditioned (1 μM), or MPP^+^ (10 μM) medium for 12 h; red, actin-tracker red. (**C**) Confocal image showing the process of wild-type (WT) and PRAG1 knock-out SH5Y cells; green, actin-tracker green. (**D**) The process length of the cells treated with rotenone were quantified; Students’ *t* test. **** *p* < 0.0001. (**E**) The viability of WT and PRAG1 knock-out cells treated with control (Ctrl) and 1 μM rotenone (Rot) medium detected via the CCK8 assay; two-way ANOVA with Sidak’s multiple comparison’s test. *** *p* < 0.0001. (**F**) Confocal images showing the cell morphology of SH-SY5Y cells transfected with EGFP-PRAG1 mutants. Then, the cells were stained with actin-tracker 555 and DAPI. (**G**) Quantification of the cell size in (**F**). * *p* < 0.05, ** *p* < 0.01, *** *p* < 0.001, **** *p* < 0.0001. One-way ANOVA with Dunnett’s multiple comparisons test. (**H**) PRAG1 knock-out SH-SY5Y cells transfected with same dose of EGFP-PRAG1 mutants were treated with control (DMSO, 1 μL/mL), rotenone-conditioned (30 μM) for 12 h, and the mortality (normalized to blank control) was assessed via the CCK8 assay. * *p* < 0.05, *** *p* < 0.001, **** *p* < 0.0001, ns, non-significant; one-way ANOVA with Dunnett’s multiple comparisons test for comparing rotenone-treated group; two-way ANOVA with Sidak’s multiple comparisons test for comparing the control group and rotenone-treated group. (**I**) SH-SY5Y cells were transfected with or without EGFP-PRAG1 plasmid and then treated with 10 μM Y-27632 or 1 μM Cytochalasin D for 12 h. Cell viability was assessed using the CCK8 assay. **** *p* < 0.01, one-way ANOVA with Dunnett’s multiple comparisons test.

Having demonstrated the role PRAG1 condensation in promoting cell contraction and observed the general formation of PRAG1 puncta under stress, we sought to explore the role of PRAG1 condensation under stress specifically under mitochondrial stress. Differential interference contrast (DIC) images revealed that treatment with rotenone or MPP^+^ induced surface blebbing and cell shrinkage (Figure 7A). Confocal imaging further confirmed that treatment with rotenone or MPP^+^ led to F-actin retraction (Figure 7B), indicating the process retraction and cell contraction, confirming mitochondrial stress induces cell contraction and process retraction [30]. To investigate the role of PRAG1 in mediating cell contraction, we generated a PRAG1 knock-out SH-SY5Y cell line (Figure S6). Our observations indicated that PRAG1 knockout did not affect normal cytoskeleton organization but inhibited rotenone-induced process retraction (Figure 7C,D). Additionally, the assessment of cell viability by the CCK8 assay revealed a protective effect of PRAG1 knockout under stress (Figure 7E). These results indicated that PRAG1-mediated cell contraction is cytotoxic.

To determine whether the condensation ability of PRAG1 is involved in mediating cell contraction, we transfected condensation-deficient PRAG1 mutants into SH-SY5Y cells. Cells expressing wild-type PRAG1 showed reduced cell size (Figure 7F,G), whereas cells expressing single-site mutants, PRAG-αN-deletion, and PRAG1-αJ deletion, showed comparable cell size to the blank control group (Figure 7F,G), suggesting attenuated cell contraction compared to cells expressing wild-type PRAG1. Rotenone treatment caused significant cell death in cells expressing wild-type PRAG1, whereas cells expressing PRAG1 mutants exhibited reduced cell death upon rotenone treatment, suggesting decreased sensitivity to rotenone. (Figure 7H). These findings suggest that PRAG1 condensation plays a negative role in the cellular response to stress by promoting cell contraction.

Y-27632, a specific Rho-kinase inhibitor, strongly suppresses cell contraction. The inhibitory effect of PRAG1 on cell contraction has also been demonstrated in a previous study [3]. To confirm that PRAG1-mediated cell contraction contributes to cell death, we investigated the effect of treatment with drugs targeting the cytoskeleton. Cytochalasin D (Cyto D) is a cell-permeable inhibitor of actin polymerization [31]. Our results show that treatment with 10 uM Y-27632 significantly rescued PRAG1-induced cell death (Figure 7I), with no noticeable effect in the control group (Figure 7I). In contrast, treatment with 1 uM Cytochalasin D not only induced cell death in control cells without PRAG1 expression (Figure 7I) but also exacerbated cell death in cells expressing PRAG1 (Figure 7I). These findings further support the involvement of PRAG1-mediated cell contraction in cell death.

**Figure 8:**
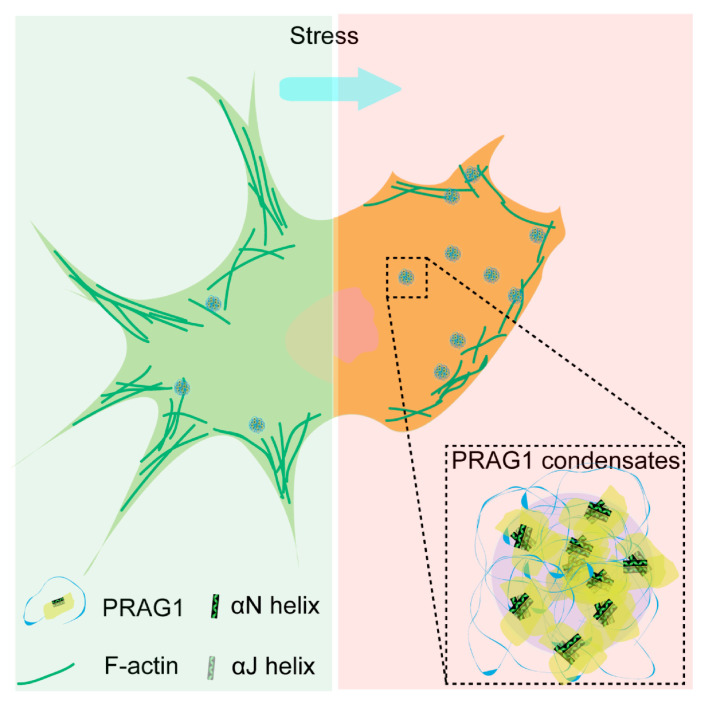
Scheme depicting the role of PRAG1-formed condensates function in promoting cell contraction and process retraction under stress.

Additionally, treatment with H_2_O_2_, SB, SA, and CCCP also resulted in a reduction in process length, surface blebbing, and cell shrinkage (Figure S7), indicating cell contraction. Given that H_2_O_2_, SB, SA, and CCCP treatment led to increased PRAG1 puncta, as mentioned above (Figure 6J,K), this suggests that the formation of PRAG1 puncta serves as a general mechanism promoting cell contraction under stress conditions (Figure 8).

## 4. Discussion

In this study, we demonstrated that PRAG1 undergoes condensation in cells, a process mediated by its αN and α-J helices. Our findings revealed that PRAG1 condensates are enriched in proteins involved in the regulation of cell morphology and adhesion. Notably, PRAG1 promotes cell contraction in a condensation-dependent manner (Figure 8). Additionally, the enhanced formation of PRAG1 puncta serves as a general response mechanism to various stressors that model human neuronal disease. These findings highlight the need for further investigation into the role of PRAG1 condensation in the context of human disease.

### 4.1. Maintaining Normal Morphology Is Crucial for Cell Survival Under Stress

Maintaining normal cell morphology is crucial for cellular survival under stress. An intact cytoskeleton is essential for various cellular processes, including endocytosis, migration, proliferation, and polarization [32,33]. The loss of cell volume and morphology during cell death is a passive process that facilitates the breakdown of the cell into smaller, apoptotic bodies [34]. Additionally, the stabilization of nascent neuronal morphology is critical for synaptic transmission [35]. In this study, we demonstrate that PRAG1 knockout leads to decreased vulnerability to stress, while PRAG1 condensation-dependent cell contraction increases stress vulnerability. Thus, PRAG1 condensate-mediated cell contraction is cytotoxic. Given the importance of maintaining normal morphology for cellular homeostasis and signaling transmission, targeting PRAG1 condensation may provide an alternative strategy to prevent cell death. PRAG1 is widely expressed in various human tissue according to human protein atlas database. PRAG1-mediated cell contraction functions as a universal mechanism in response to different stressors. Therefore, exploring whether PRAG1 condensation could serve as a promising therapeutic target for human disease is of great importance. Additionally, we detected the preferential expression of PRAG1 in vulnerable DA neuron clusters. PRAG1 puncta were found to be significantly upregulated in rotenone and MPP^+^-treated cells, as well as in DA neurons derived from PD patients. These findings suggest that PRAG1 condensation may be involved in process retraction and synaptic impairment in neurodegenerative diseases like PD. Due to technical limitations, we were unable to generate cell lines with in situ GFP-labeled PRAG1 at endogenous levels. Thus, we temporarily termed antibody-stained PRAG1 structures as “puncta” Further studies are needed to determine whether these puncta, observed at endogenous expression levels in assay experiments, exhibit dynamics similar to those of EGFP-PRAG1 condensates in future studies.

### 4.2. Role of PRAG1 in Promoting Cell Contraction

PRAG1 has been described as a catalytically inactive protein kinase [9] and as an effector of the small GTPase RND2, which stimulates RhoA activity and inhibits NGF-induced neurite outgrowth [3]. Additionally, PRAG1 promotes Src family kinase (SFK) signaling by regulating the subcellular localization of CSK, a negative regulator of these kinases, thereby regulating cell morphology and motility [6]. Building on these findings and our results, we confirm that PRAG1 contributes to cell death by promoting cell contraction. Importantly, we are the first to identify the condensation capability of PRAG1 and its role in promoting cell contraction under various stress conditions.

Rho family GTPases are known to regulate the cytoskeleton and morphological changes [6]. Rnd2 binds to PRAG1 in a GTP-dependent manner, subsequently activating RhoA and inducing cell contraction [3]. However, we did not observe Rnd2-forming condensates [3], nor did our proteomic analysis reveal RND2 enrichment in PRAG1 condensates. Nevertheless, we clearly observed colocalization of PRAG1 and RhoA in our cell model. Given that PRAG1 condensates cluster around F-actin bundles, it appears that PRAG1 induces cell contraction. Previous studies have also demonstrated that PRAG1 overexpression alone is sufficient to induce cell contraction [3]. Accordingly, we propose that PRAG1-formed condensates recruit RhoA to stimulate its activity, leading to cell contraction. However, the mechanism driving PRAG1 condensation and recruitment to PRAG1 condensates require further investigation. Furthermore, the function of other molecules enriched in PRAG1 also need further exploration.

### 4.3. Identification of the IDRs Using Different Algorithms

Disordered regions are characterized as entire proteins or specific protein segments that lack a fixed three-dimensional structure. Due to their highly dynamic nature, these regions adopt a range of conformations, which make it challenging to determine their precise structure using techniques such as cryo-EM or other structural determination methods. Over 50 disorder predictors, based on various algorithms with distinct principles, have been developed by researchers [36]. However, the accuracy of these tools can vary significantly due to differences in their underlying algorithms and methodologies. The development of IDR predictors began with small collections of disordered proteins and IDRs determined by various methods. The amino acid sequence is used as inputs, and the predictors give disorderliness scores as outputs. Due to the variability of input proteins and the algorithms and parameters set by different researchers from different labs, different computational models lead to different predictions of protein disorders.

Also, the prediction results from a set of individual predictors can serve as inputs for another predictor, known as a meta-predictor. This approach often leads to a significant improvement in prediction accuracy, as the different predictors contribute unique information derived from diverse sequence features, prediction models, and training datasets [36]. PONDR (VLXT, artificial neural networks-based) [37], PONDR (VL3-BA, also annotated as VL3, artificial neural networks-based) [37], and IUPred3 (based on a biophysics-based model) [38] are all meta-predictors because they are derived from combinations of individual predictors. Thus, PONDR (VLXT), PONDR (VL3), and IUPred3 algorithms, which have been widely used in recent studies [10,11,12,13,14], were used in this study for disorderliness predication. The AA116-458 and AA458-944 regions of PRAG1, which exhibit continuity, were annotated as “disordered regions” by the VLXT and VL3 algorithms. These results showed similarity with the predictions from IUPred3, leading us to select these two IDRs for further investigation. Based on our imaging results, neither the AA116-458 and AA458-944 regions nor the αN and αJ helices deletion mutants formed condensates comparable to those of full-length PRAG1. These findings suggest that the condensation activity of PRAG1 is mediated by the coordinated interplay between its disordered and ordered region.

### 4.4. PRAG1 Condensates and PRAG1 Dimers

PRAG1 is known to form homodimers with itself through its C-terminus, and PRAG1 also forms heterodimers with PEAK1 (pseudopodium-enriched atypical kinase 1, also called SGK269) [8]. Both homodimers and heterodimers rely on interactions mediated by αN and αJ helices located at its C-terminus [2,6]. Our findings indicate that the condensation ability of PRAG1 also depends on its αN and αJ helices, highlighting the complexity of its intermolecular interactions and condensation ability. The dimerization of PRAG1 is crucial for its role in maintaining cellular bioprocess by regulating protein tyrosine phosphorylation [6]. However, the precise function of the SGK269/PRAG1 heterodimer remains unclear. In this study, we observed that mCherry-tagged SGK269 does not form condensates and is diffusely distributed within cells (Figure S6B). Notably, co-expression of PRAG1 and SGK269 significantly inhibited the formation of PRAG1 condensates (Figure S6B). This suggests that the transition between dimer and PRAG1 condensates may be regulated by its interaction with SGK269. Furthermore, the dimerized PRAG1 or PRAG1/SGK269 heterodimer may play a non-negligible role under normal conditions. It is noteworthy that PRAG1 knock-out cells maintain normal morphology, similar to wild-type cells, and show less perturbation in their morphology and F-actin network, as well as reduced sensitivity to stress. Thus, PRAG1 condensates serve as a novel mechanism in response to stress, distinct from dispersed PRAG1. Further studies are needed to elucidate the underlying mechanism regulating PRAG1 condensation and cell contraction dynamics.

### 4.5. Limitations

In this study, we investigated the dynamic properties of PRAG1 condensates using FRAP, fusion assay, 1,6-hexanediol perturbation, and Cry2 labeling. These approaches support the formation of PRAG1 condensates through phase separation. However, the inability to purify PRAG1 protein for in vitro LLPS assays represents a limitation, as such experiments would provide direct evidence of LLPS-driven condensate formation. Moreover, the current Cry2-labeling strategy also displayed a negative effect on the dynamic properties of the PRAG1 condensates. To address this, further optimization of tag proteins, linker proteins, and buffer solutions is required to enable successful protein purification. Additionally, improved labeling strategies would facilitate the generation of cell lines with endogenously labeled PRAG1, allowing for in situ investigations on its role in regulating cell contraction under stress conditions.

## 5. Conclusions

This study aimed to investigate the role of PRAG1 condensation under stress conditions. We not only uncovered the dynamic nature of PRAG1-formed condensate, but also revealed their critical role in promoting cell contraction under various stress conditions. These findings provide novel insights into the mechanisms of cell contraction and the bahavior of membraneless condensates in response to stress. Future studies will focus on validating these results at endogenous level and in animal models to elucidate the physiological relevance of PRAG1 condensation. nly uncovered the dynamic nature of PRAG1-formed condensate, but also revealed their critical role in promoting cell contraction under various stress conditions. These findings provide novel insights into the mechanisms of cell contraction and the bahavior of membraneless condensates in response to stress. Future studies will focus on validating these results at endogenous level and in animal models to elucidate the physiological relevance of PRAG1 condensation.

## Data Availability

Further information and requests for resources and reagents should be directed to and will be fulfilled by the Lead Contact, Wei Yang (yangwei@zju.edu.cn).

## Supplementary Materials

The following supporting information can be downloaded at https://www.mdpi.com/article/10.3390/biom15030379/s1: Figure S1: PRAG1 is widely expressed in human tissues; Figure S2: PRAG1 forms condensates in cells; Figure S3: Sequence alignment of PRAG1 across human, mouse and rat; Figure S4: Light-induced condensation assay reveals the physical properties of optoPRAG1 condensates; Figure S5: PRAG1 independently forms condensates; Figure S6: Verification of PRAG1 knock-out SH-SY5Y cell line; Figure S7: Various stressors induce cell contraction.

## Abbreviations

The following abbreviations are used in this manuscript:

PRAG1	Peak1-related, kinase-activating pseudokinase 1
IDR	Intrinsic disordered region
LLPS	Liquid–liquid phase separation
Dopamine	DA
PONDR	Prediction of natural disordered region
optoPRAG1	CRY2-mCherry-PRAG1
Rot	Rotenone
iPSC	Induced pluripotent stem cell
PD	Parkinson’s disease
MPP^+^	N-Methyl-4-Phenylpyridinium^+^
SA	Sodium arsenite
SB	D-sorbitol
CCCP	Carbonyl cyanide 3-chlorophenylhydrazone
H_2_O_2_	Hydrogen peroxide
LB	Lewy body
SNc	Substantia nigra pars compacta
PFF	Pre-formed fibrils
SG	Stress granule
CytoD	Cytochalasin D
