# Supplementary material for: PRAG1 Condensation Drives Cell Contraction Under Stress
Source: Biomolecules. 2025 Mar 5;15(3):379. doi: 10.3390/biom15030379 (PMC11939857; doi:10.3390/biom15030379)
Supplement: Supplementary file 1 [file biomolecules-15-00379-s001.zip › biomolecules-3453280-supplementary.pdf]

PRAG1 condensation drives cell contraction

Peiwu Ye, Peiran Jian, Luyu Ye, Min Liu, Qiuyuan Fang, Peilin Yu, Jianhong Luo, Huanxing Su, Wei Yang

Corresponding authors: Huanxing Su (huanxingsu@umac.mo), Wei Yang (yangwei@zju.edu.cn)

This file includes:

Figure legends of supplementary figures S1-S7.

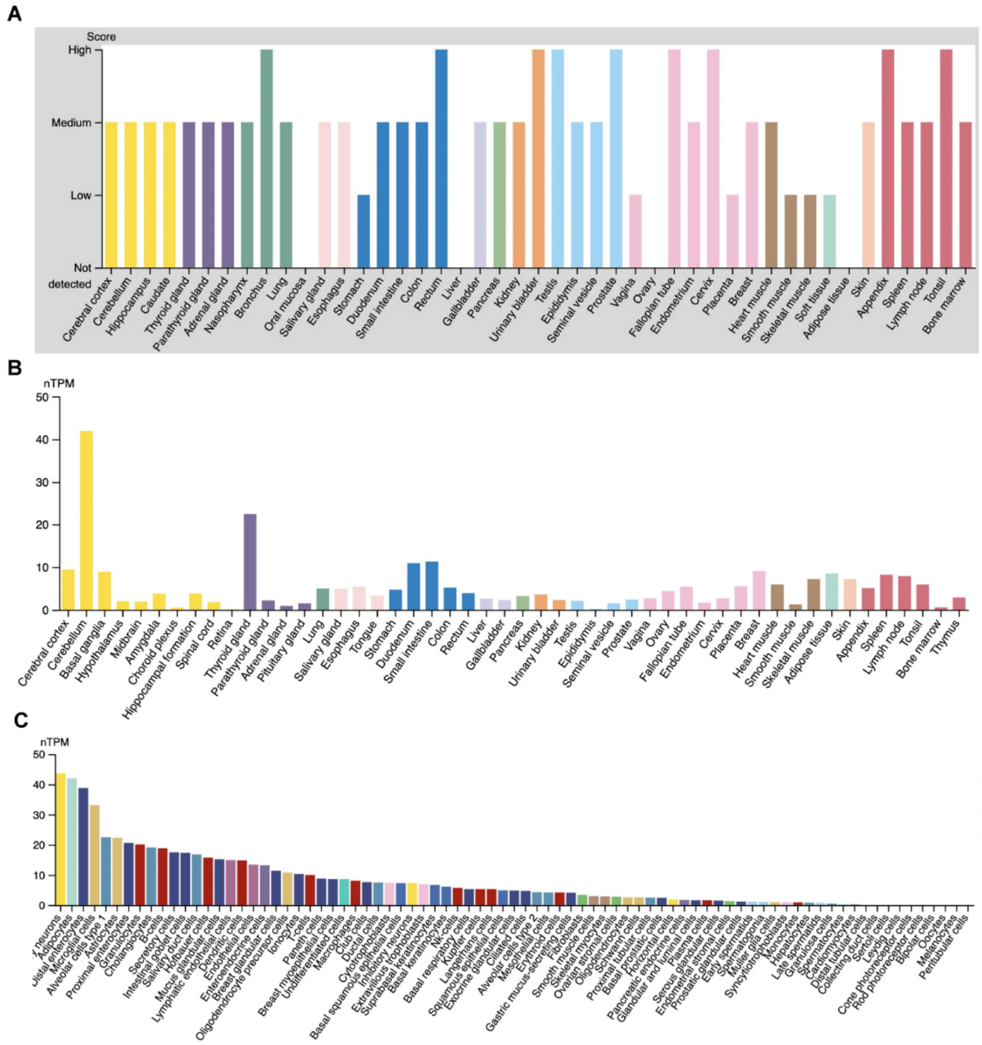

Figure S1. Expression data derived from human protein atlas (HPA) showing PRAG1 is widely expressed in human.

(A) Histogram showing the protein expression overview of human PRAG1. Data source: https://www.proteinatlas.org/ENSG00000275342-PRAG1/tissue.

(B) Histogram showing the RNA expression overview of human PRAG1. Data source: https://www.proteinatlas.org/ENSG00000275342-PRAG1/tissue.

(C) Histogram showing the RNA single cell type specificity of human RPAG1. Data source: https://www.proteinatlas.org/ENSG00000275342-PRAG1 /single+cell#single_cell_type_summary.

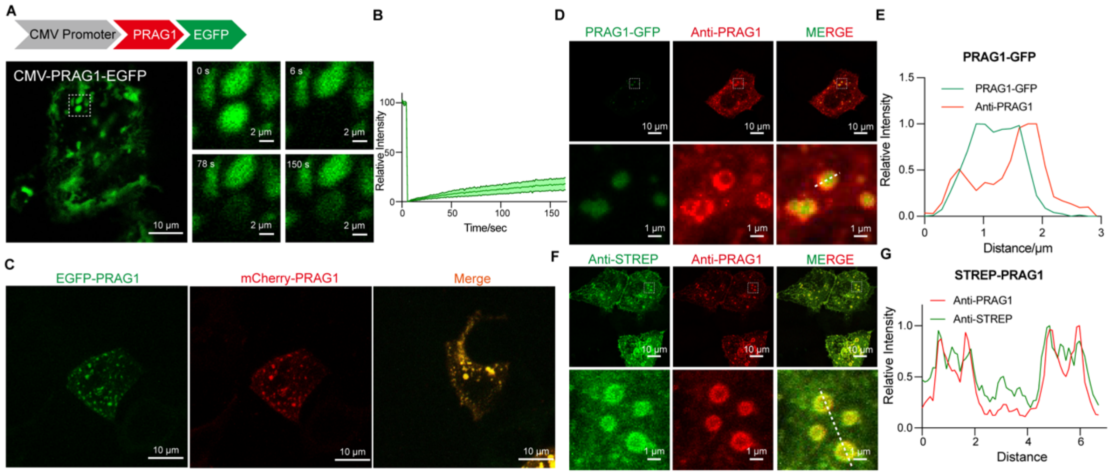

Figure S2. PRAG1 forms condensates in cells.

(A) Confocal images of representative SH-SY5Y cells expressing PRAG1-EGFP. PRAG1-EGFP protein was diffusely distributed throughout the cell body with punctate structures. Fluorescence recovery after photobleaching (FRAP) showed dynamic property of PRAG1-EGFP puncta.

(B) FRAP analysis of PRAG1-EGFP puncta. n = 7.

(C) Confocal images of representative SH-SY5Y cells co-expressing EGFP- PRAG1 and mcherry-PRAG1. Scale bar, 10 μm.

(D) SH-SY5Y cells expressing PRAG1-EGFP were stained with anti- PRAG1antibody.

(E) Line scan showing the related intensity profiles of exogenous-transfected EGFP-PRAG1with the signal of antibody stained PRAG1.

(F) SH-SY5Y cells expressing STREP-PRAG1 were stained with anti- PRAG1 antibody.

(G) Line scan showing the related intensity profiles of exogenous-transfected STREP-PRAG1 puncta (stained with anti-STREP antibody) with the signal of stained PRAG1.

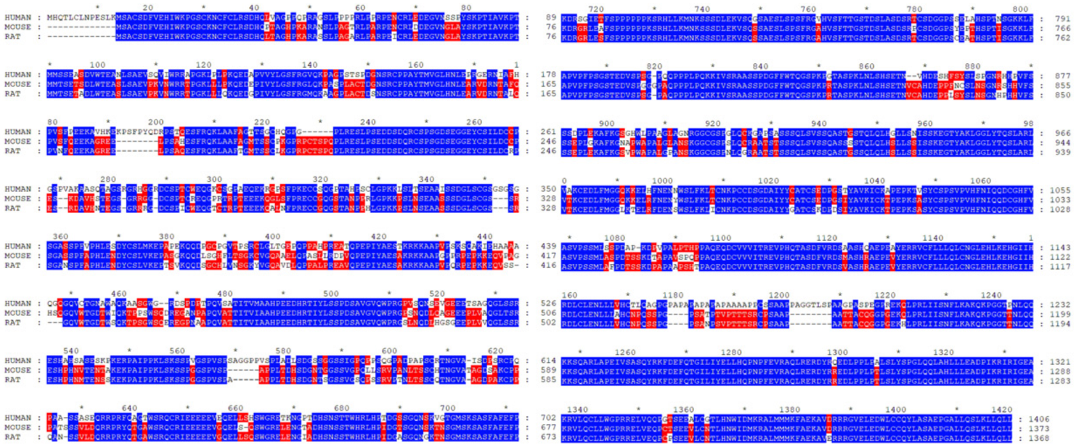

Figure S3. PRAG1 is conserved in human, mouse and rat.

Sequence alignment of PRAG1 across human, mouse and rat.

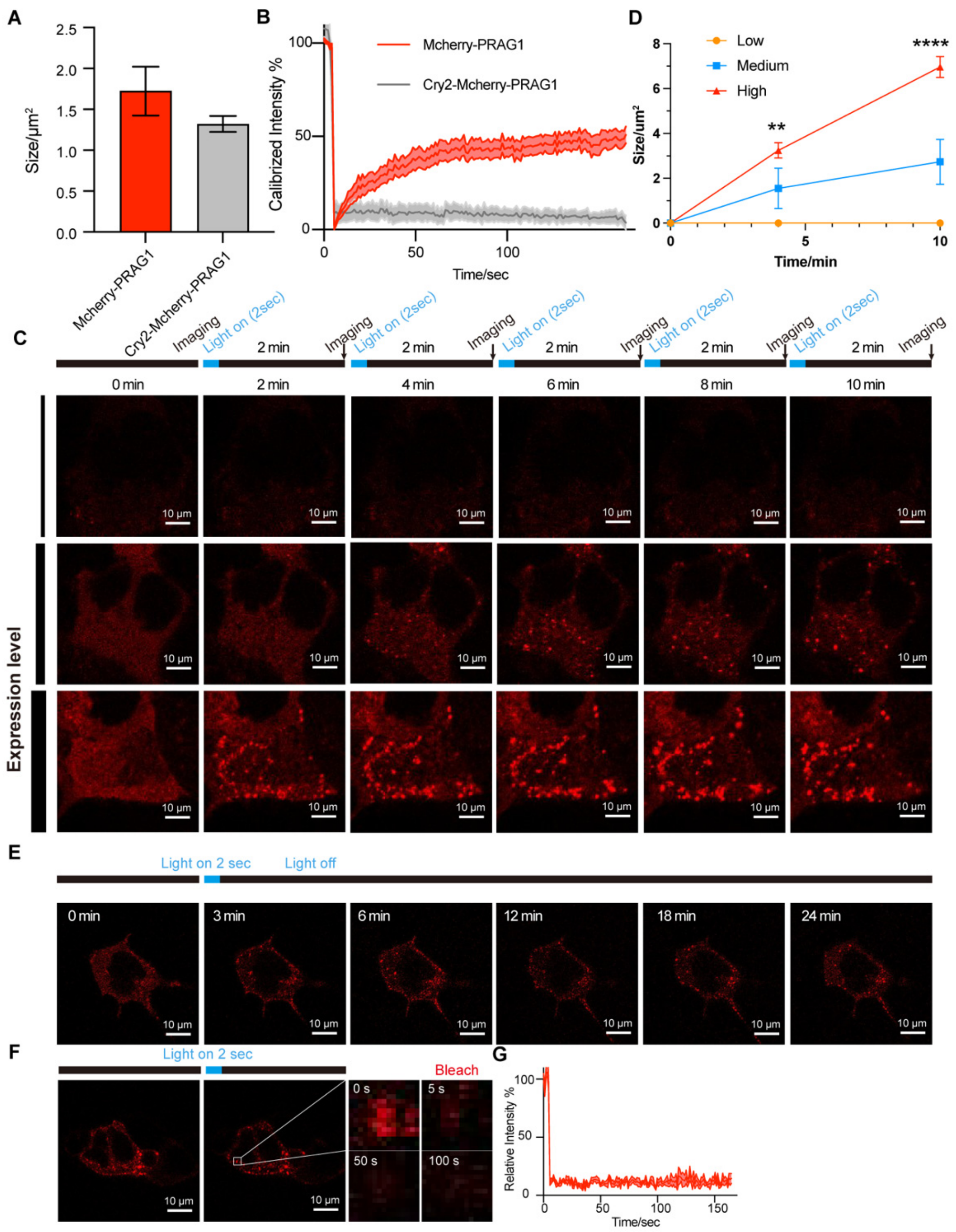

Figure S4. Light-induced condensation assay reveals the physical properties of optoPRAG1 condensates.

(A) Quantification of the size of condensates formed by Mcherry-PRAG1 and Cry2-Mcherry-PRAG1. n = 27, 26. Students' t test.

(B) FRAP analysis of condensates formed by Mcherry-PRAG1 and Cry2-Mcherry-PRAG1. n = 9, 5.

(C) Images showing SH-SY5Y cells with varying expression level of were exposed to blue light (5%).

(D) Quantification of the size of light-induced optoPRAG1 condensates. $^{**}p < 0.01$, $^{****}p < 0.0001$, Two-way ANOVA with Sidak's multiple comparisons test.

(E) Images showing cells with optoPRAG1 expression were stimulated with a series of blue light (5%).

(F) Images showing light-induced optoPRAG1 condensates were bleached.

(G) FRAP analysis of light-induced optoPRAG1. $n = 4$.

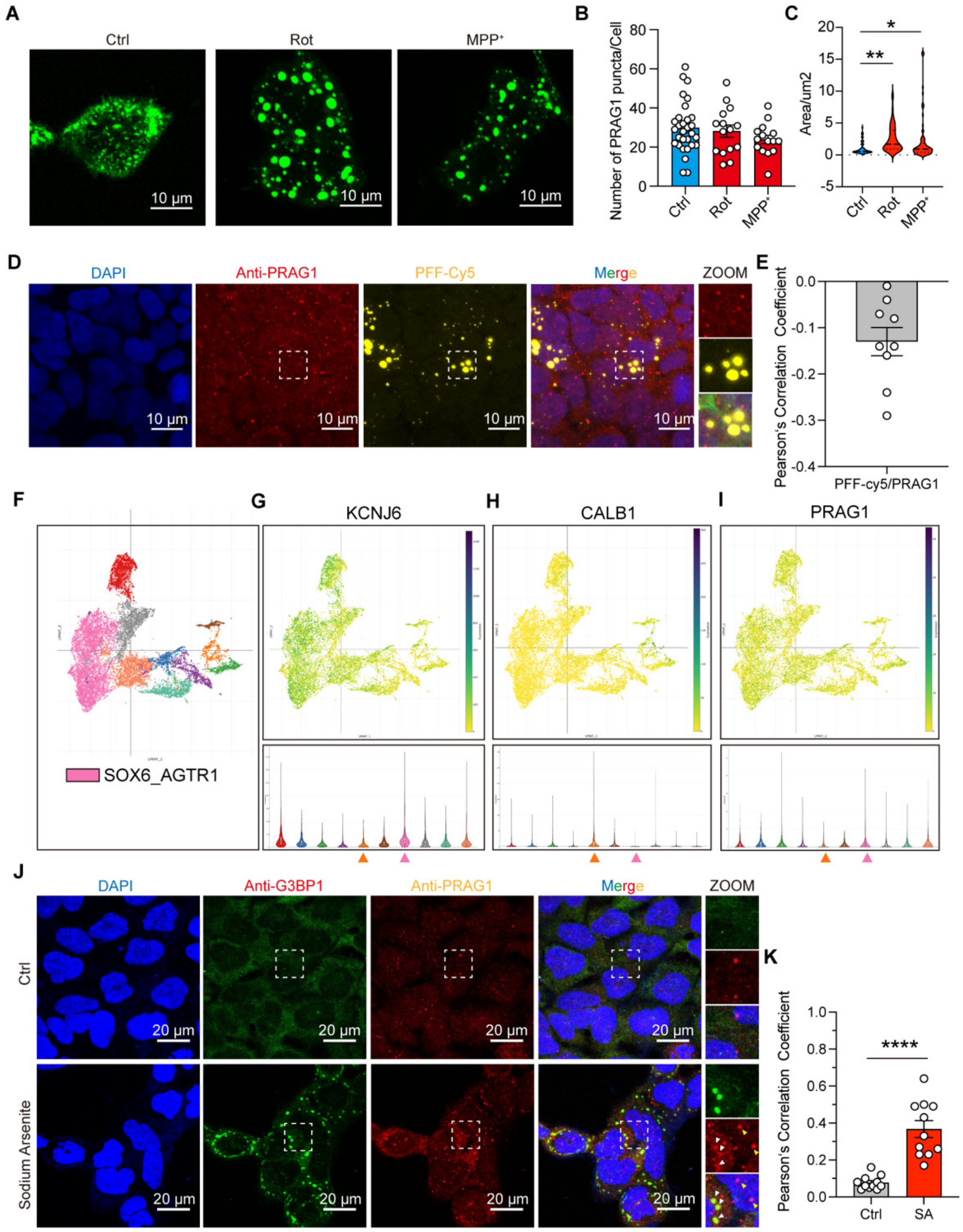

Figure S5. PRAG1 independently forms condensates

(A) Images showing cells with EGFP-PRAG1 expression were exposed to 1 μM rotenone and 10 μM MPP^+ for 12 hrs.

(B) The number of EGFP-PRAG1 condensates per cell in (A) were quantified. n = 30, 15, 15, respectively. One-way ANOVA with Dunnett's multiple comparisons test.

(C) The size of EGFP-PRAG1 condensates after rotenone and MPP^+ treatment was quantified. n = 52, 61, 80, respectively. One-way ANOVA with Dunnett's multiple comparisons test. *$p < 0.05$, **$p < 0.01$.

(D) 10 μM PFF-cy5 was added to SH-SY5Y cells for 12 hrs. Then the cells were stained with anti-PRAG1 antibody (Red).

(E) Pearson's correlation coefficient analysis of PFF-Cy5 with PRAG1 in panel (A).

(F) UMAP displaying the SOX6_AGTR1 positive DA neuron cluster from postmortem human SNc.

(G) Upper panel, scatterplot depicting the expression pattern of KCNJ6 gene. Lower panel, boxplot showing the expression level of KCNJ6 in different neuron clusters.

(H) Upper panel, scatterplot depicting the expression pattern of CALB1. Lower panel, boxplot showing the expression level of CALB1 in different neuron clusters.

(I) Upper panel, scatterplot depicting the expression pattern of PRAG1. Lower panel, boxplot showing the expression level of PRAG1 in different neuron clusters.

(J) Confocal images showing the distribution of endogenous PRAG1 puncta and stress granule (marked by G3BP antibody) in SH-SY5Y cells. The cells were treated with control medium or 0.5 μM sodium arsenate (SA) for 0.5 hr.

(K) Pearson's correlation coefficient showing significant enhanced co-localization of PRAG1 puncta with stress granules (marker protein: G3BP1). Student's t test. ****p < 0.0001.

Data source of single-nucleus RNA-sequencing of DA neurons from postmortem human SNc:

https://singlecell.broadinstitute.org/single_cell/study/SCP1768/single-cell-genomic-profiling-of-human-dopamine-neurons-identifies-a-population-that-selectively-degenerates-in-parkinsons-disease-single-nuclei-data.

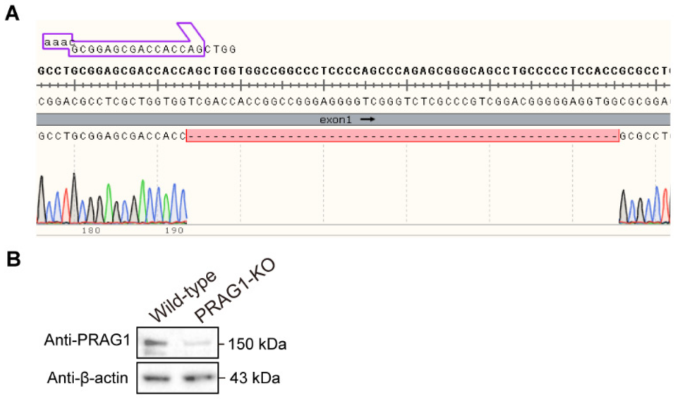

Figure S6. Verification of PRAG1 knock-out in SH-SY5Y cell line

(A) Genome sequencing reveals that sgRNA targeting exon 1 of PRAG1 results in 52 bp deletion.

(B) The expression of PRAG1 in wild-type and PRAG1-KO cell lines were determined by western blotting.

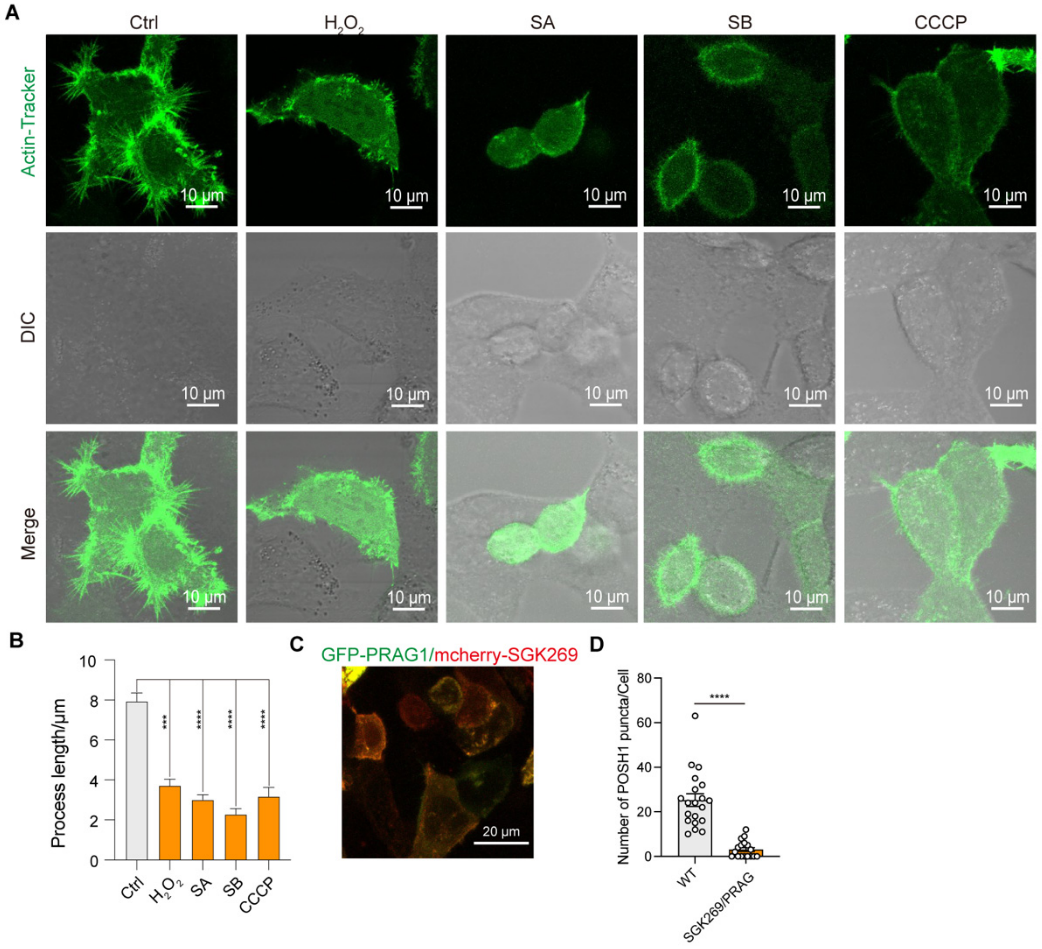

Figure S7. Diverse stressors induce cell contraction

(A) Representative confocal and DIC images showing the effect of 1 mM hydrogen peroxide (H_2O_2), 0.4 M D-sorbitol (SB), 0.5 μM sodium arsenate (SA), and 10 μM CCCP treatment for 30 min on cell morphology in SH-SY5Y cells.

(B) Quantification of the process length in (A). ***$p < 0.001$, ****$p < 0.0001$. One-way ANOVA with Dunnett's multiple comparisons test.

(C) Representative confocal images showing the distribution of GFP-PRAG1 and mcherry-SGK269 in SH-SY5Y cells.

(D) Quantification of the number of EGFP-PRAG1 condensates per cell with or without SGK269 co-expression. n = 20, 19, ****$p < 0.0001$, Students' t test.

Original blot images

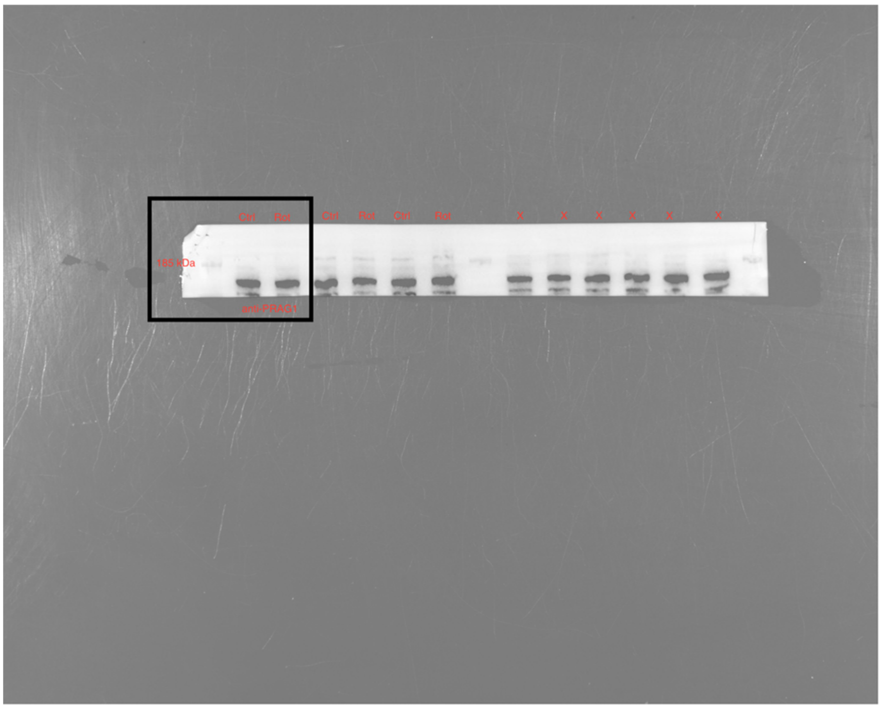

(A) Related to Figure 6D: Original Western blot images showing the expression of **human PRAG1 in SH-SY5Y cells following** g **1 μM rotenone treatment for 12 hrs**. Lane 1 and lane 8: molecular weight marker of 185 kDa (11832124, Thermo Fisher Scientific Inc., Waltham, MA, USA). Lanes 2-7: Control and rotenone-treated groups, lane 1 and lane 2 (marked with a black box) were cropped and used as the representative image in Figure 6D. The irrelevant samples were marked with "X". The blot was probed with anti-PRAG1 antibody (HPA012066, 1:1000; MilliporeSigma, Burlington, MA, USA), followed by horseradish peroxidase-conjugated AffiniPure goat anti-rabbit IgG (A0208, 1:5000; Beyotime Biotechnology, Shanghai, China). The expression level of PRAG1 was normalized to β-actin (shown in panel (B)). Students' t test.

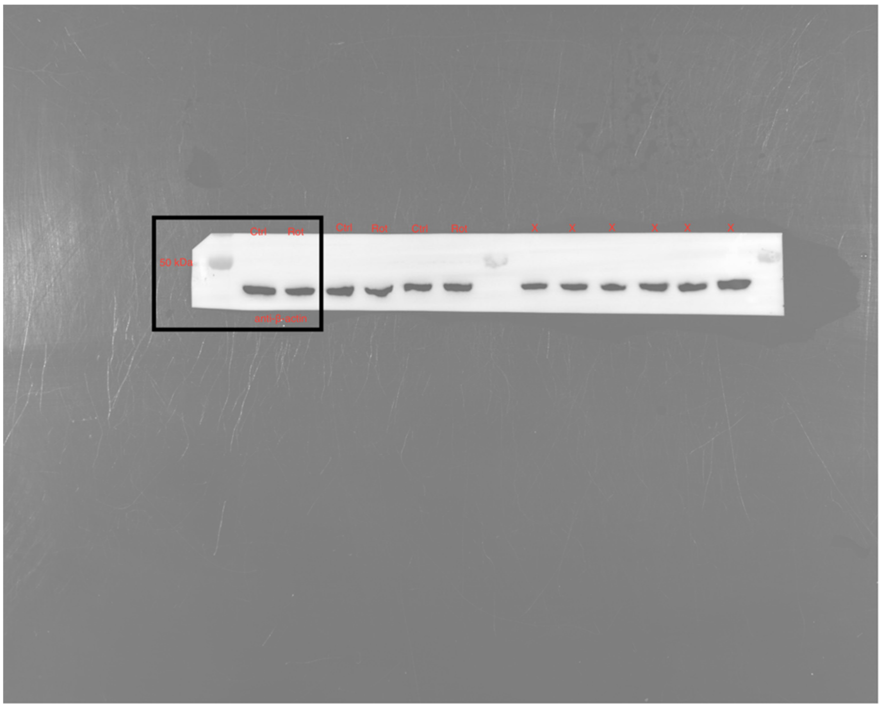

(B) Related to Figure 6D: Original Western blot images showing the expression of **human β-actin in SH-SY5Y cells following 1 μM rotenone treatment for 12 hrs**. Lane 1 and lane 8: molecular weight marker of 50 kDa (11832124, Thermo Fisher Scientific Inc., Waltham, MA, USA). Lanes 2-7: Control and rotenone-treated groups, lane 1 and lane 2 (marked with a black box) were cropped and used as the representative image in Figure 6D. The irrelevant samples were marked with "X". The blot was probed with anti-β-actin antibody (AC026, 1:2000; ABclonalTechnology, Woburn, MA, USA), followed by horseradish peroxidase-conjugated AffiniPure goat anti-rabbit IgG (A0208, 1:5000; Beyotime Biotechnology, Shanghai, China).

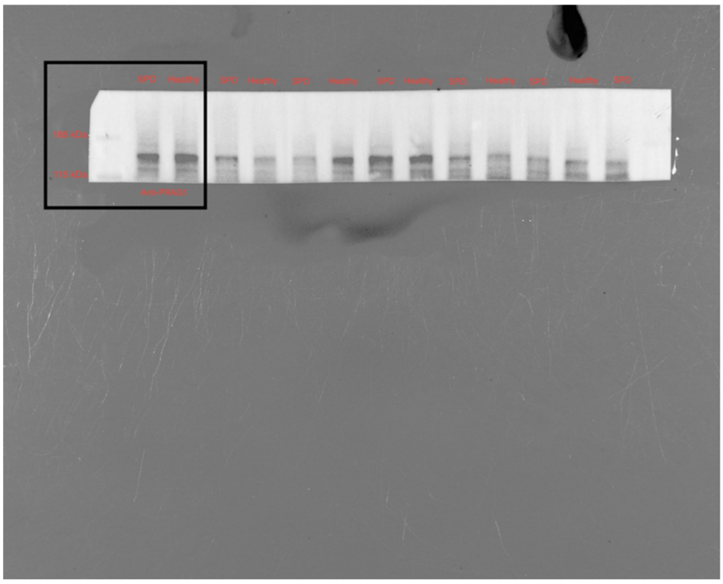

(C) Related to Figure 6H: Original Western blot images showing the expression of **human PRAG1 in iPSC-differentiated DA neurons.** Lane 1 and lane 15: molecular weight markers of 115 and 185 kDa (11832124, Thermo Fisher Scientific Inc., Waltham, MA, USA). Lanes 2-14: PD patient (SPD) and healthy groups, lane 1 and lane 2 (marked with a black box) were cropped and used as the representative image in Figure 6H. The blot was probed with anti-PRAG1 antibody (HPA012066, 1:1000; MilliporeSigma, Burlington, MA, USA), followed by horseradish peroxidase-conjugated AffiniPure goat anti-rabbit IgG (A0208, 1:5000; Beyotime Biotechnology, Shanghai, China). The expression level of PRAG1 was normalized to β-actin (shown in panel (D)). Students' t test.

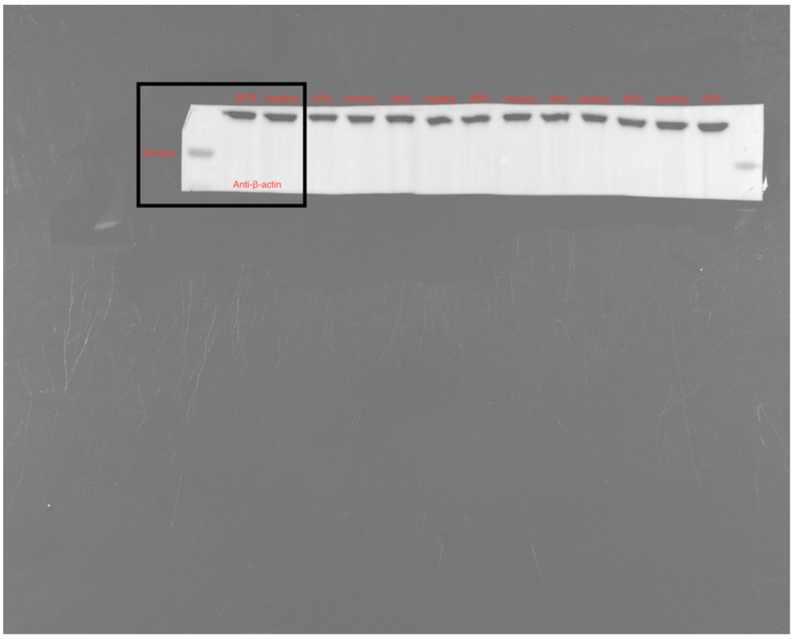

(D) Related to Figure 6H: Original Western blot images showing the expression of **human β-actin in iPSC-differentiated DA neurons.** Lane 1 and lane 15: molecular weight marker of 30 kDa (11832124, Thermo Fisher Scientific Inc., Waltham, MA, USA). Lanes 2-14: PD patient (SPD) and healthy groups, lane 1 and lane 2 (marked with a black box) were cropped and used as the representative image in Figure 6H. The blot was probed with anti-β-actin antibody (AC026, 1:2000; ABclonalTechnology, Woburn, MA, USA), followed by horseradish peroxidase-conjugated AffiniPure goat anti-rabbit IgG (A0208, 1:5000; Beyotime Biotechnology, Shanghai, China).

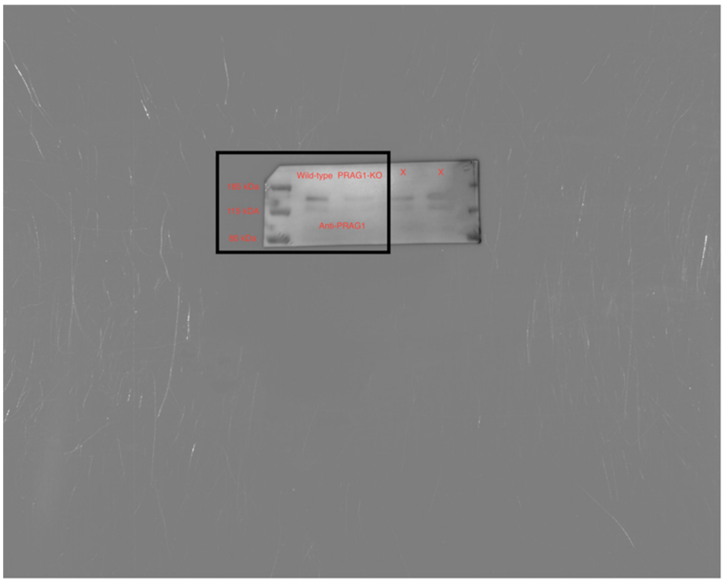

(E) Related to Figure S6B: Original Western blot images showing the knockout efficiency of **human PRAG1 in SH-SY5Y cells**. Lane 1: molecular weight markers of 80, 115, 185 kDa (11832124, Thermo Fisher Scientific Inc., Waltham, MA, USA). Lanes 2-3: wild-type and PRAG1 knockout cell lines (marked with a black box), lane 2 and lane 3 were cropped and used as the representative image in Figure S6B. The irrelevant samples were marked with "X". The blot was probed with anti-PRAG1 antibody (HPA012066, 1:1000; MilliporeSigma, Burlington, MA, USA), followed by horseradish peroxidase-conjugated AffiniPure goat anti-rabbit IgG (A0208, 1:5000; Beyotime Biotechnology, Shanghai, China).

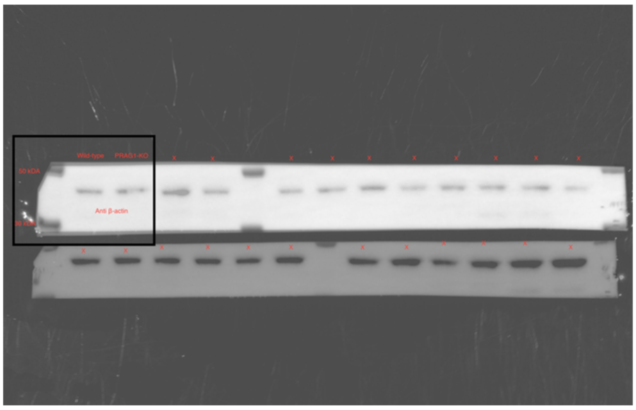

(F) Related to Figure S6B: Original Western blot images showing the expression of **human β-actin in SH-SY5Y cells after PRAG1 knockout**. Lane 1: molecular weight markers of 30 and 50 kDa (11832124, Thermo Fisher Scientific Inc., Waltham, MA, USA). Lanes 2-3: wild-type and PRAG1 knockout cell lines (marked with a black box), lane 2 and lane 3 were cropped and used as the representative image in Figure S6B. The irrelevant samples were marked with "X". The blot was probed with anti-β-actin antibody (AC026, 1:2000; ABclonalTechnology, Woburn, MA, USA), followed by horseradish peroxidase-conjugated AffiniPure goat anti-rabbit IgG (A0208, 1:5000; Beyotime Biotechnology, Shanghai, China).
